# Supplementary material for: Stable hydrogen evolution reaction at high current densities via designing the Ni single atoms and Ru nanoparticles linked by carbon bridges
Source: Nat Commun. 2024 Mar 12;15:2218. doi: 10.1038/s41467-024-46553-9 (PMC10933429; doi:10.1038/s41467-024-46553-9)
Supplement: Supplementary file 1 — Supplementary Information [file 41467_2024_46553_MOESM1_ESM.pdf]

Stable hydrogen evolution reaction at high current densities via designing the Ni single atoms and Ru nanoparticles linked by carbon bridges

Rui Yao[1,6], Kaian Sun[2,6], Kaiyang Zhang[1], Yun Wu[1], Yujie Du[1], Qiang Zhao[1], Guang Liu[1*], Chen Chen[3], Yuhan Sun[4,5*]& Jinping Li[1,4*]

[1]College of Chemical Engineering and Technology, Shanxi Key Laboratory of Gas Energy Efficient and Clean Utilization, Taiyuan University of Technology, Taiyuan 030024, China.

[2]College of Materials Science and Engineering, Fuzhou University, Fuzhou 350108, China..

[3]Department of Chemistry, Tsinghua University, Beijing 100084, China.

[4] Shanxi Research Institute of Huairou Laboratory, Taiyuan 030031, China.

[5]2060 Research Institute, Shanghai Tech University, Shanghai 201210, China.

[6]These authors contributed equally

Table of Contents

1. Supplementary Figures 1-60

2. Supplementary Notes 1-28

3. Supplementary Tables 1-11

4. Supplementary References

Supplementary Figures

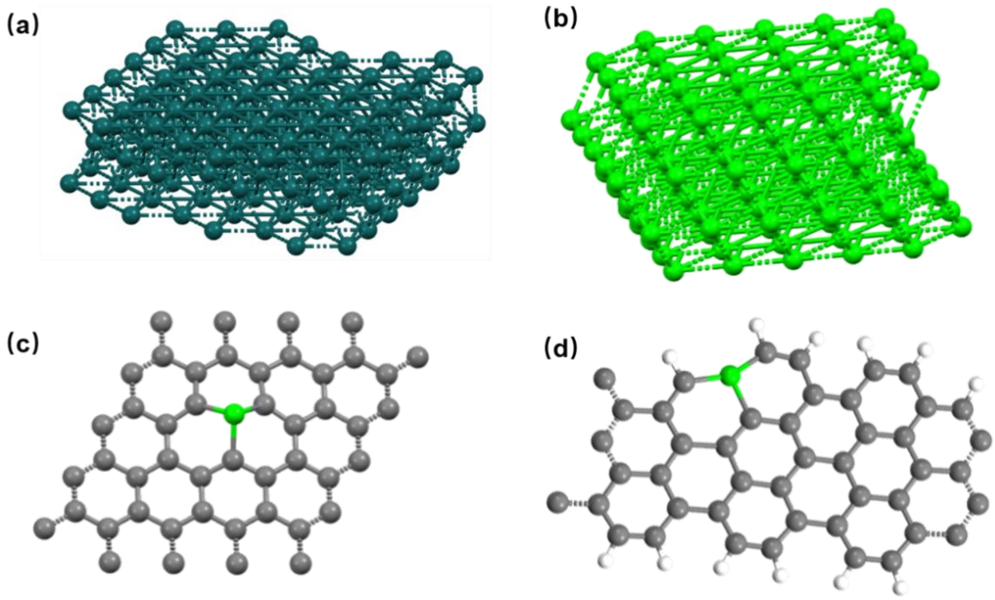

Supplementary Fig. 1 | Theoretical calculation models. a, Ru (002), **b**, Ni (111), **c**, Ni_{sub}/C and (d) Ni_{def}/C.

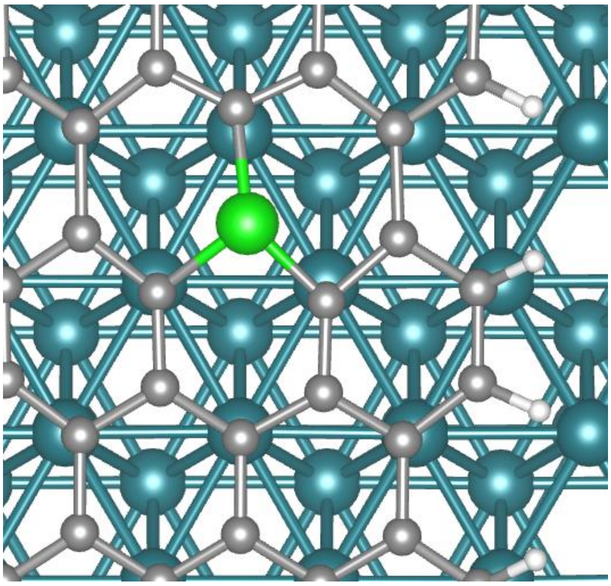

Supplementary Fig. 2 | Theoretical calculation model of $RuNi_{sub}/C$.

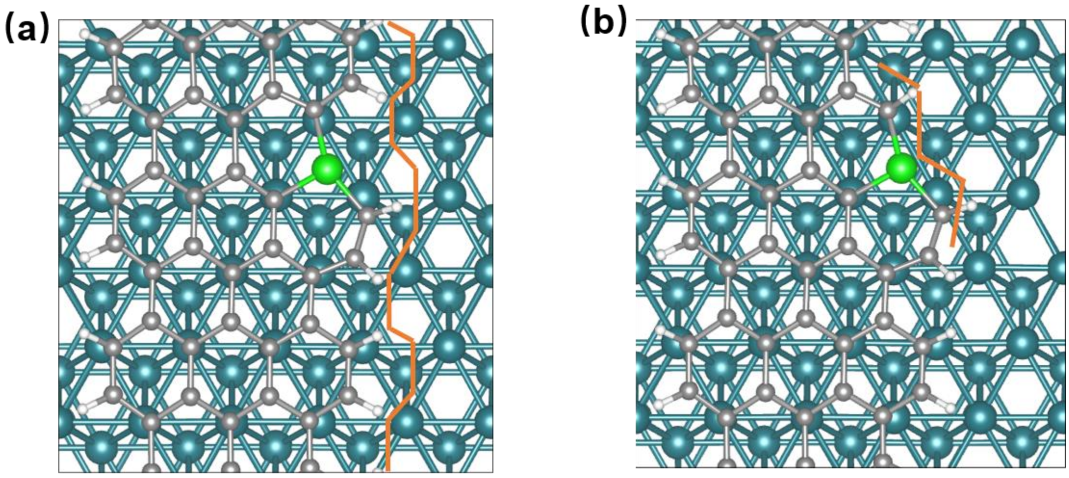

Supplementary Fig. 3 | Theoretical calculation models of RuNi$_{def/}$C with two typical structures for the graphene edge. a, armchair models, **b**, zigzag models.

Supplementary Note 1 | The formation energies of the RuNi$_{def/}$C with armchair-like and zigzag-like graphene edge are both -3.36 eV, indicating that the two models are equally stable.

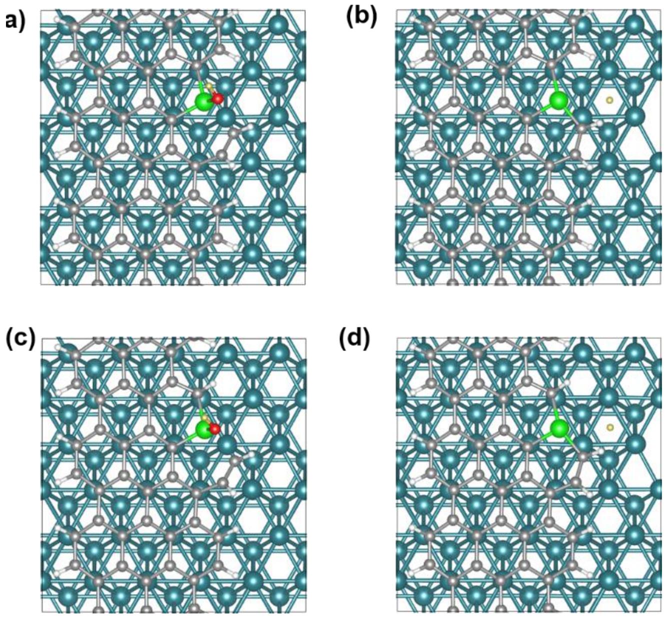

Supplementary Fig. 4 | Adsorption of OH and H intermediates for $RuNi_{def/}C$ with **a, b** armchair-like and **c, d** zigzag-like graphene edge.

Supplementary Note 2 | The OH* adsorption energies (E_{OH*}) of the $RuNi_{def/}C$ with armchair-like graphene edge (A-$RuNi_{def/}C$) and zigzag-like graphene edge (Z-$RuNi_{def/}C$) are 0.42 eV and 0.39 eV, respectively, indicating that both have similar adsorption capacities for OH*, whereas the ΔG_{H*} of the corresponding two models are -0.18 and -0.15, indicating that zigzag-like graphene edge has an advantage from this point of view.

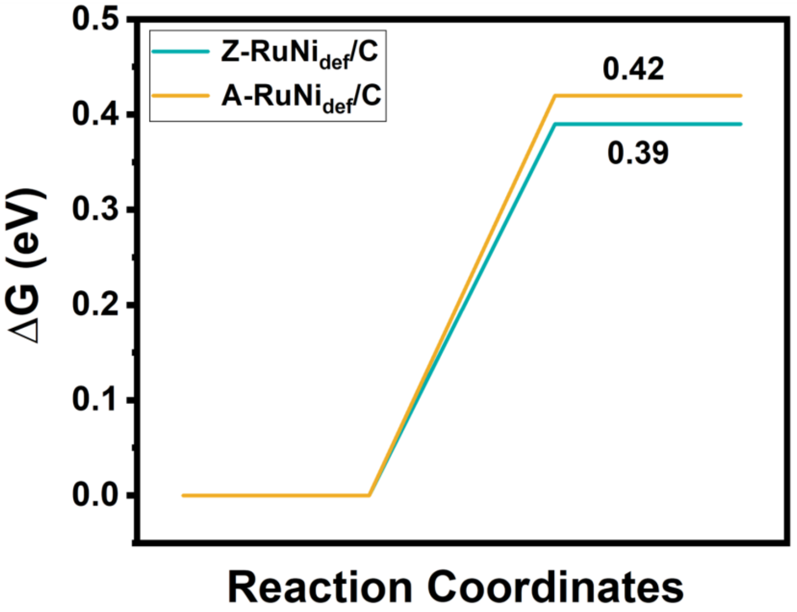

Supplementary Fig. 5 | Partial reaction coordinates for HER process for A-RuNi$_{def/}$C and Z-RuNi$_{def/}$C in order to screen graphene edge types.

Supplementary Note 3 | Apparently, RuNi$_{def/}$C with zigzag-like graphene edge has a lower energy barrier for the water decomposition transition state, so the RuNi$_{def/}$C with zigzag-like graphene edge was used for a series of subsequent studies. It is noteworthy that the energy barrier for water decomposition transition state, OH* adsorption, and ΔG_{H*} of RuNi$_{def/}$C with both armchair-like and zigzag-like graphene edge are superior to those of pure Ni$_{def}$ and Ru (002).

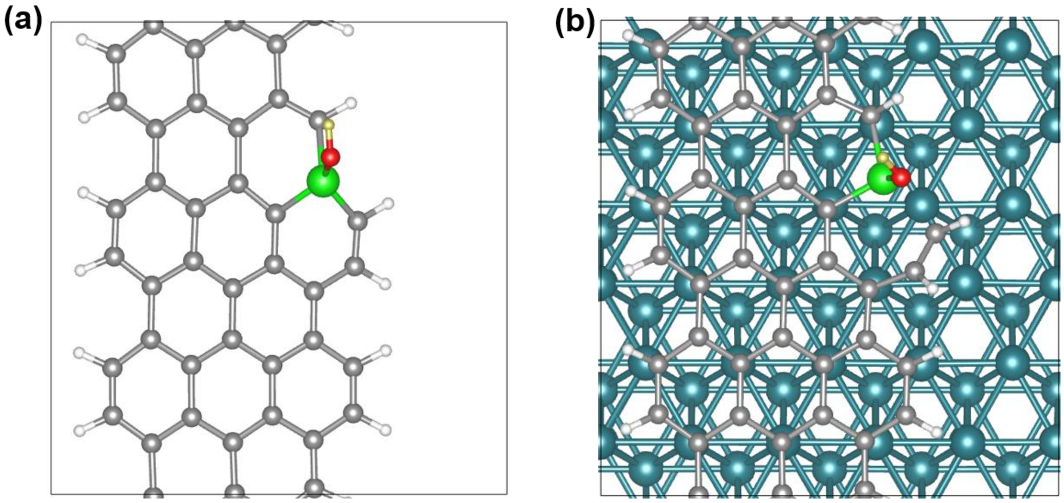

Supplementary Fig. 6 | Adsorption of OH intermediates on the Ni site for **a**, Z-$Ni_{def/}$C and **b,** Z-$RuNi_{def/}$C.

Supplementary Note 4 | The OH* adsorption energies (E_{OH*}) on the Ni site for Z-$Ni_{def/}$C and Z-$RuNi_{def/}$C are 0.32 eV and 0.42 eV, respectively, indicating that the introduction of Ru site weakened the adsorption of OH at the Ni site due to the interaction between Ru and Ni.

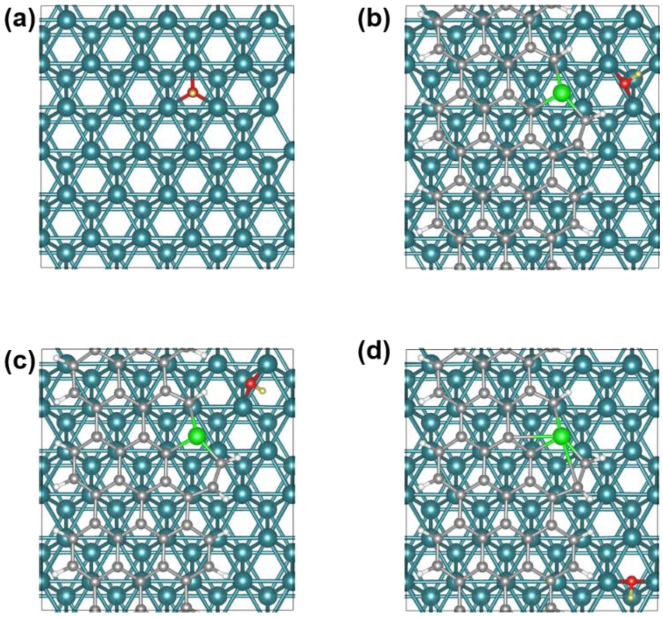

Supplementary Fig. 7 | OH intermediates is adsorbed on the Ru site with the adsorbed Ru is located at different distances from Ni : **a,** Ru (002), **b**, near position ($RuNi_{def/}$C-N), **c**, middle position ($RuNi_{def/}$C-M) and **d,** far position ($RuNi_{def/}$C-F).

Supplementary Note 5 | The OH* adsorption energies (E_{OH*}) on the Ru site for Ru (002), $RuNi_{def/}$C-N, $RuNi_{def/}$C-M and $RuNi_{def/}$C-F are -0.43 eV, -0.15 eV, -0.18 eV and -0.23 eV, respectively, indicating that the adsorption of OH at the Ru site is weakened due to the interaction between Ru and Ni, and the closer the distance between Ru and Ni, the stronger this weakening effect is. It is worth noting that the best model in this manuscript is $RuNi_{def/}$C-N.

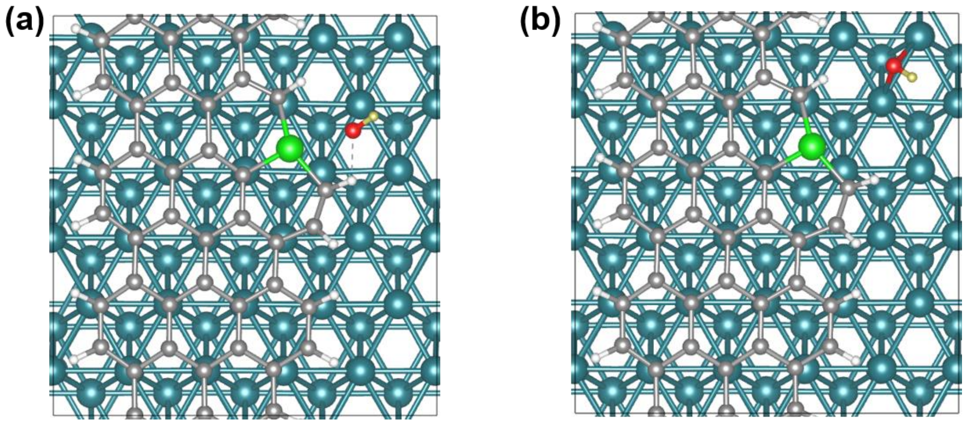

Supplementary Fig. 8 | a, Initial and **b**, final states for the OH adsorbed on the Ni-Ru bridge site.

Supplementary Note 6 | When OH adsorbs is adsorbed at the Ni-Ru bridging site, the end state of adsorption remains at the Ru site due to the strong adsorption capacity of Ru. Therefore, the bridging site for OH adsorption is equivalent to the Ru site.

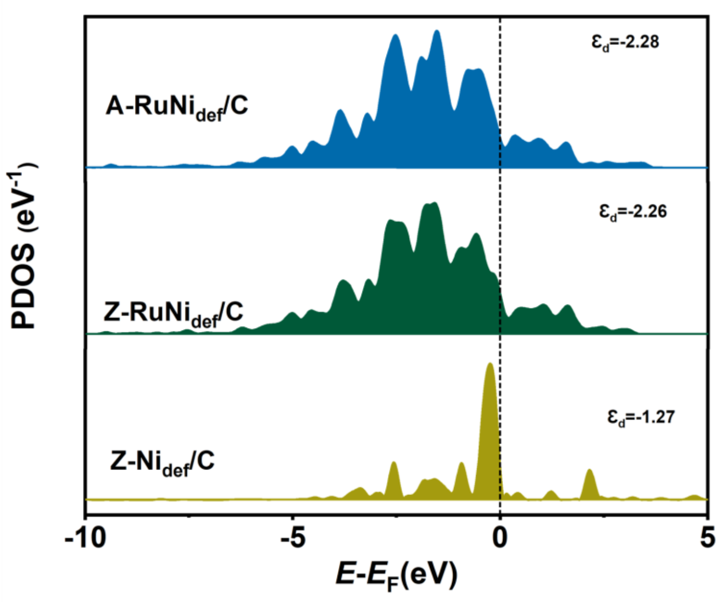

Supplementary Fig. 9 | PDOS of Ni for Ni$_{def}$/C and RuNi$_{def}$/C with armchair-like (A-RuNi$_{def}$/C) and zigzag-like graphene edge (Z-RuNi$_{def}$/C).

Supplementary Note 7 | The d-bands centers of the two models (A-RuNi$_{def}$/C and Z-RuNi$_{def}$/C) are very close.

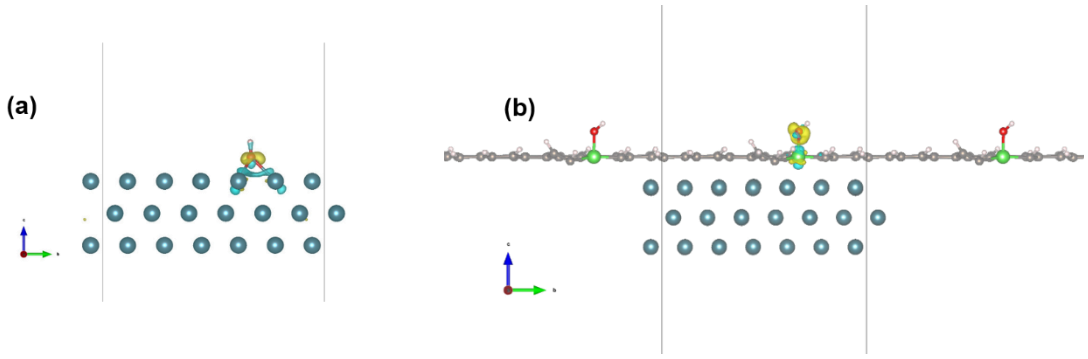

Supplementary Fig. 10 | OH differential charge density calculation results. **a**, Ru (002) and **b**, $RuNi_{def}/C$ models.

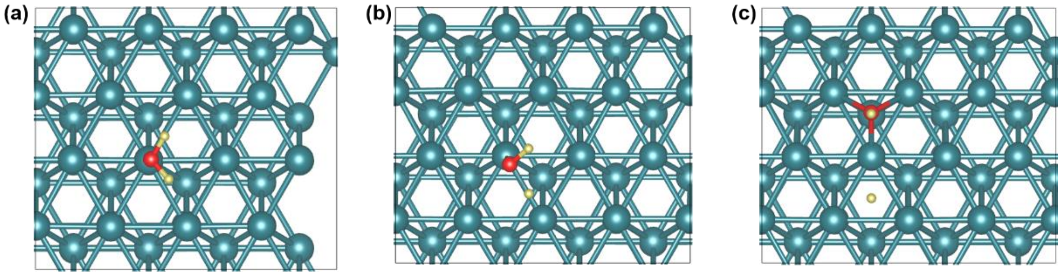

Supplementary Fig. 11 | a, Initial state, **b**, transition state and **c**, final state of HER reaction process for Ru (002) model under alkaline conditions.

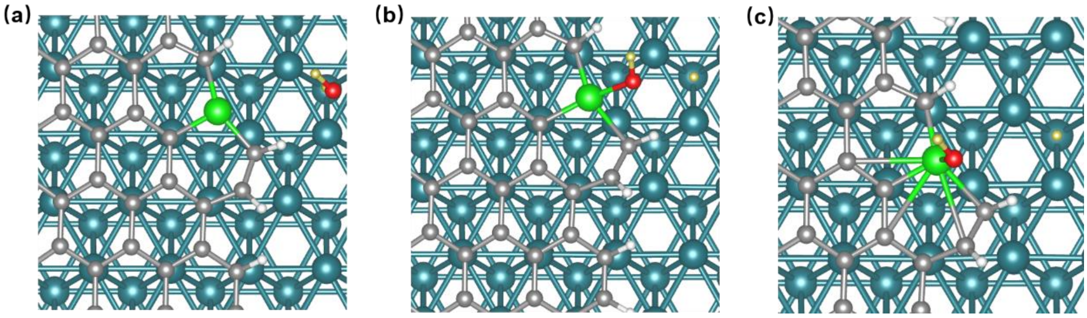

Supplementary Fig. 12 | a, Initial state, **b**, transition state and **c**, final state of HER reaction process for $RuNi_{def}/C$ model under alkaline conditions, in which the color yellow indicates H atom in H_2O, and the color white signifies H atom on the edge, and the color green, deep blue, red and grey indicates nickel, ruthenium, oxygen and carbon separately.

Supplementary Note 8 | As can be seen from the Supplementary Fig. 4, the water molecules are first adsorbed on Ru sites. In the transition state, OH intermediates are preferentially adsorbed on Ni sites.

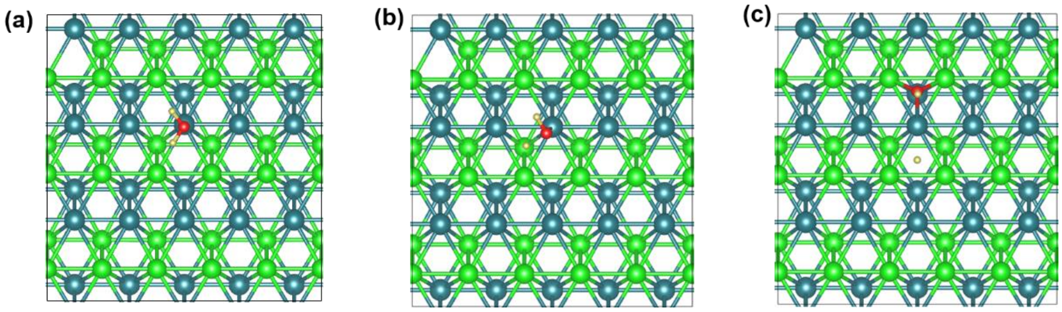

Supplementary Fig. 13 | a, Initial state, **b**, transition state and **c**, final state of HER process for RuNi alloy model under alkaline conditions, in which the color yellow indicates H atom in H_2O, and the color green, deep blue and red indicates nickel, ruthenium and carbon separately.

Supplementary Note 9 | In order to compare with the $RuNi_{def}/C$ model, the RuNi alloy with Ni partially replacing Ru crystals (002) was modeled and its water decomposition energy barrier was calculated. Particularly, the distance between the Ni and the Ru of $RuNi_{def}/C$ is about 3 Å, whereas the distance between the Ni and Ru atoms in RuNi alloy (where Ni-Ru bonds are present) is about 2 Å, which confirms that the Ni-Ru bonds are also absent in $RuNi_{def}/C$ DFT model.

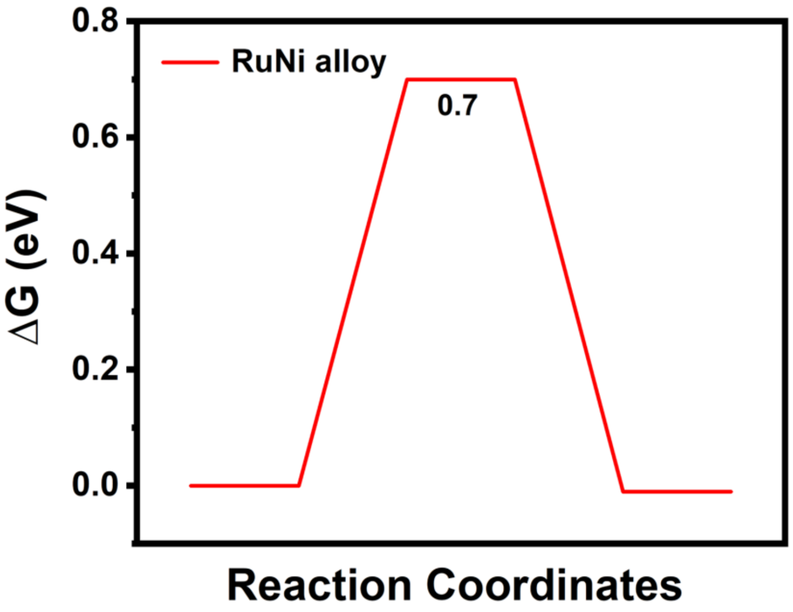

Supplementary Fig. 14 | Reaction coordinates for HER process for RuNi alloy.

Supplementary Note 10 | The RuNi alloy has a high transition state energy barrier 0.7 eV, even higher than 0.52 eV of pure Ru (002).

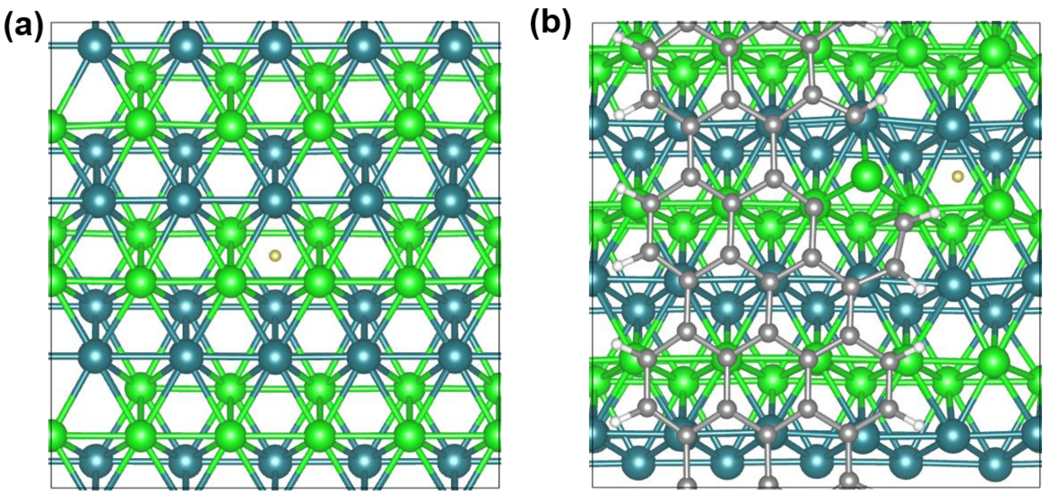

Supplementary Fig. 15 | Adsorption of H intermediates for **a,** RuNi alloy and **b,** RuNi/C with Ni replaces carbon edge, in which the color yellow, green, deep blue and grey indicates H atom in H_2O, nickel, ruthenium and carbon separately.

Supplementary Note 11 | The H* adsorption energies (ΔG_{H*}) of the RuNi alloy and RuNi/C are 0.43 eV and 0.34 eV, respectively, indicating that the interaction of RuNi alloys with Ni_{def}/C optimizes the ΔG_{H*} on the alloy.

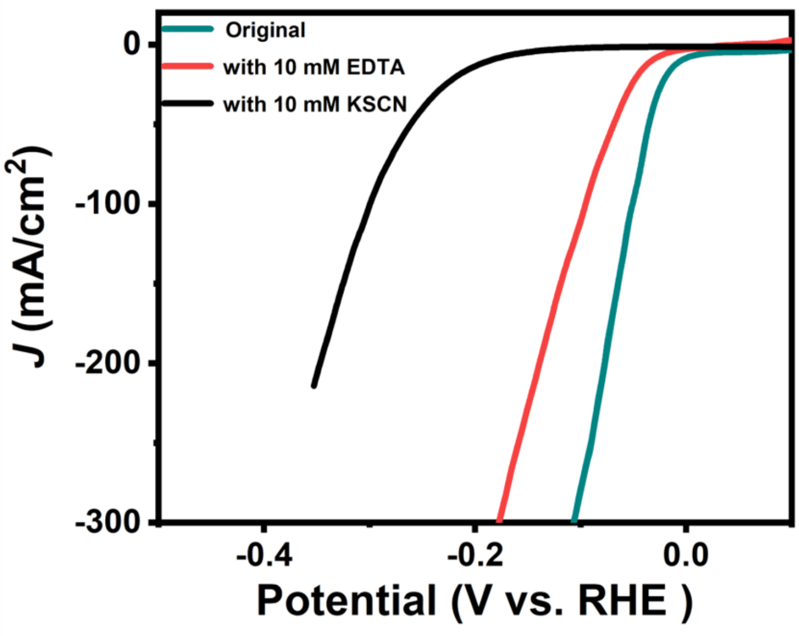

Supplementary Fig. 16 | LSV curves of UP-$RuNi_{SAs}$/C in 1 M KOH and with the addition of 10 mM EDTA or 10 mM KSCN.

Supplementary Note 12 | To distinguish the actual contribution of each species in this reaction system, we performed poisoning experiments, in which the ethylenediaminetetraacetic acid (EDTA) and potassium thiocyanate (KSCN) acid were added to the original 1.0 M KOH electrolyte respectively as complexing reagents. EDTA mainly coordinates with single atoms, while KSCN can complex with nanoparticles and single atoms to deactivate them. As shown in Supplementary Fig. 5, upon adding EDTA to the electrolyte, the HER activity of UP-$RuNi_{SAs}$/C has decreased compared with that in the pristine electrolyte without EDTA, with the overpotential increased from 9 mV to 35 mV at 10 mA cm^{-2}. However, the activity of UP-$RuNi_{SAs}$/C was significantly decreased with introduction of 10 mM KSCN into the original electrolyte, with the overpotential increased from 9 mV to 189 mV at 10 mA cm^{-2}. Such obviously difference fully proved that Ru nanoparticles made a major contribution as main active species in UP-$RuNi_{SAs}$/C.

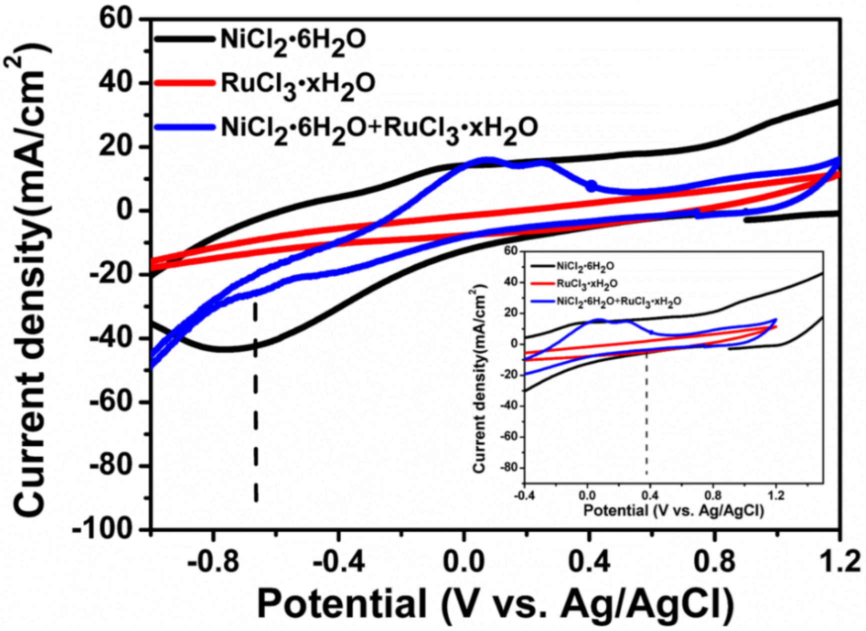

Supplementary Fig. 17 | CV of the electrolyte containing $NiCl_2 \cdot 6H_2O$, $RuCl_3 \cdot xH_2O$, and $NiCl_2 \cdot 6H_2O$+ $RuCl_3 \cdot xH_2O$ on a CFP electrode for electrodeposition.

Supplementary Note 13 | For the electrodeposition of the coatings, the region of reduction is significant for determining the range of deposition potentials. We could roughly determine from Supplementary Fig. 13 that the deposition potentials of Ni and Ru were about -0.65 V vs. Ag|AgCl and 0.4 V vs. Ag|AgCl according to the positions of their reduction peaks, thus the Ru was firstly deposited on carbon fiber paper (CFP).

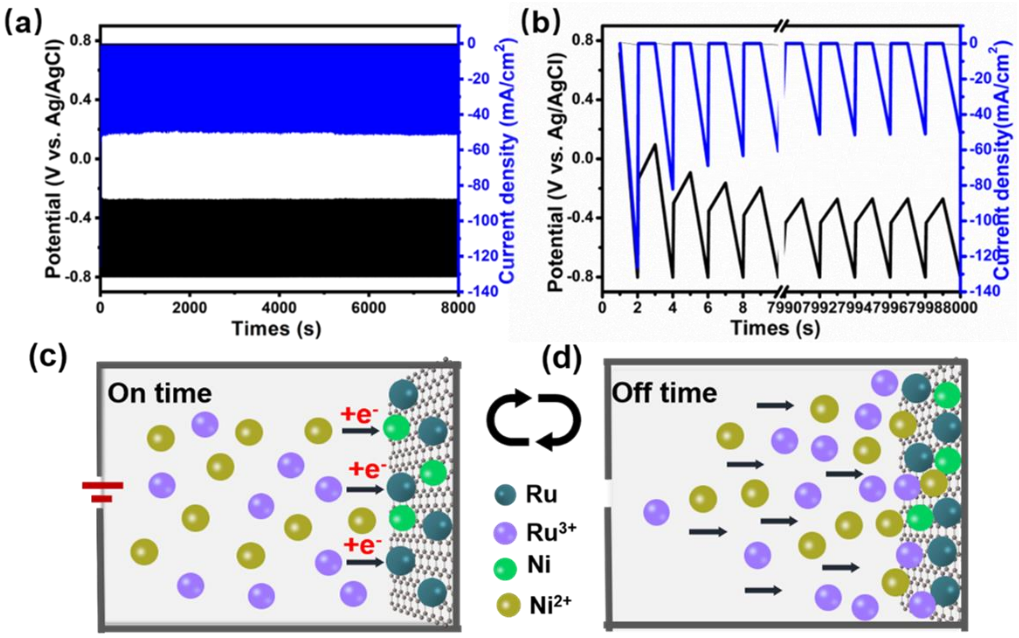

Supplementary Fig. 18 | Potential and current changes over time in the preparation of UPED method **a-b**, and **c-d** schematic of the UPED process for the fabrication of the UP-$RuNi_{SAs}$/C.

Supplementary Note 14 | For the electrodeposition of UPED process, the deposition of Ni and Ru coexisted in the form of the competitive affiliation when the deposition potential was -0.8 V vs. Ag|AgCl, of which the initial stage of deposition is more conducive to the deposition of Ru. As shown in Supplementary Fig. 18c-d, when the current is conducted (on time), Ru^{3+} is preferentially reduced to produce the concentration polarization effect, and when the current is disconnected (off time), the metal ions are replenished near the electrode in the form of diffusion, weakening the concentration polarization.

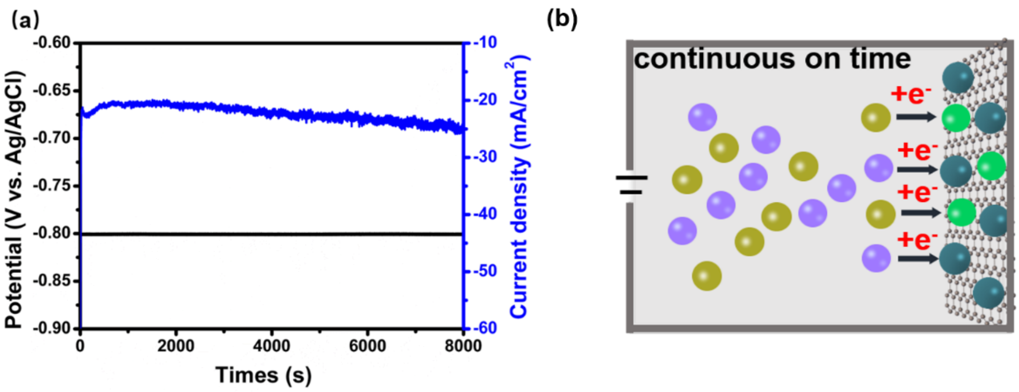

Supplementary Fig. 19 | a, Potential and current changes over time in the preparation of chronopotentiometry electrodeposition method and **b**, schematic of the chronopotentiometry electrodeposition process for the fabrication of the CP-RuNi/C.

Supplementary Note 15 | For the electrodeposition of CP process under the same potential condition (-0.8 V vs. Ag|AgCl) in Supplementary Fig. 19, the overpotential generated by the continuous Ru consumption caused by the continuous "on time" (no "off time") makes the original potential (-0.8 V vs. Ag|AgCl) increasingly unfavorable for Ru deposition, thus Ni is deposited onto the coating earlier than UPED.

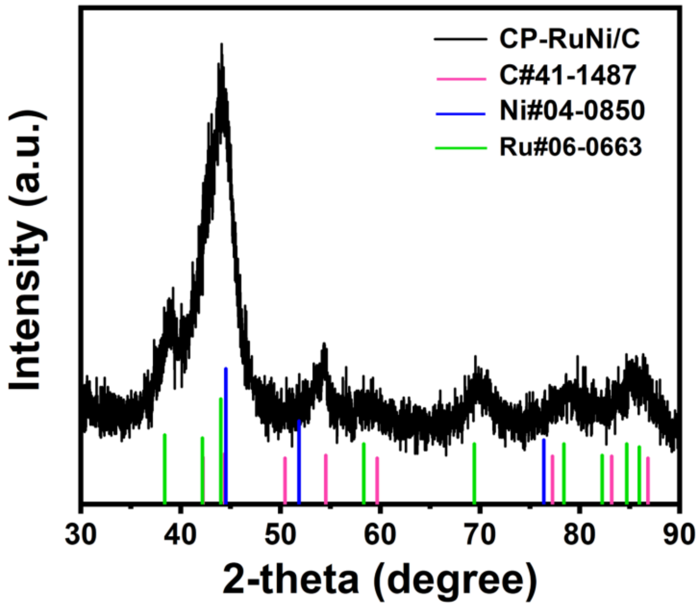

Supplementary Fig. 20 | Slow scanning XRD patterns of CP-RuNi/C.

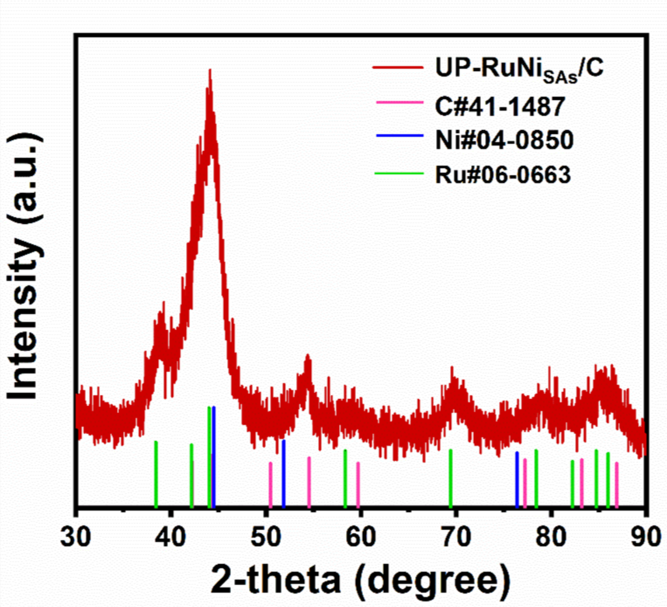

Supplementary Fig. 21 | Slow scanning XRD patterns of UP-RuNi$_{SAs}$/C.

Supplementary Note 16 | Slow scanning XRD patterns of the UP-RuNi$_{SAs}$/C catalyst only detected the partial diffraction peak of the Ru crystal.

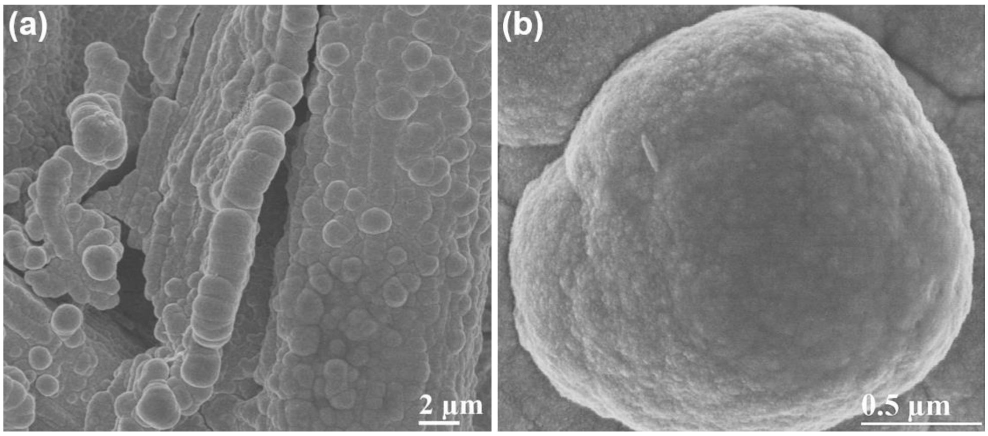

Supplementary Fig. 22 | a, b SEM image of UP-$RuNi_{SAs}$/C.

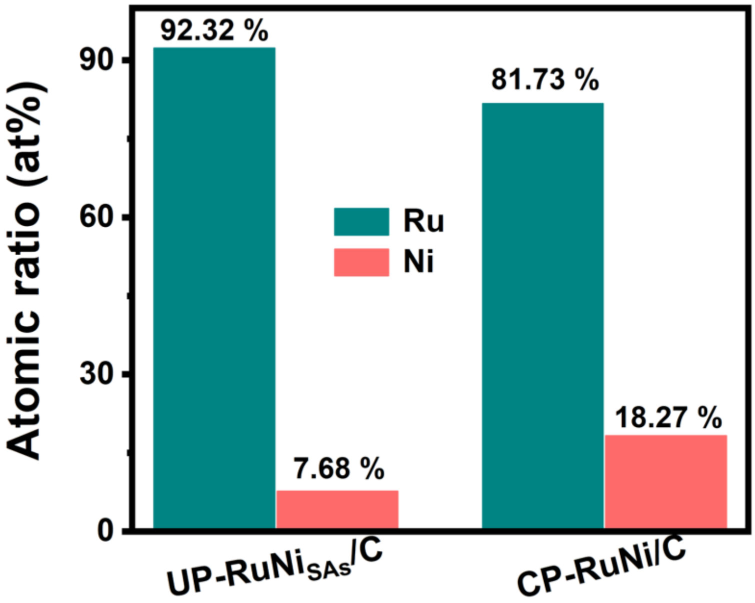

Supplementary Fig. 23 | ICP-OES analysis of UP-RuNi$_{SAs}$/C and CP-RuNi/C.

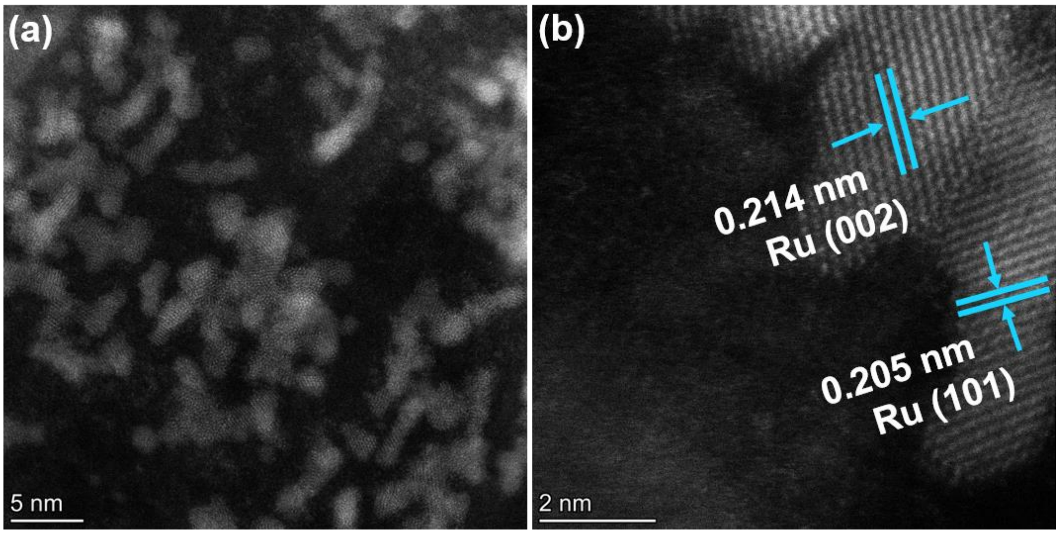

Supplementary Fig. 24 | a, b, Spherical aberration correction STEM of UP-$RuNi_{SAs}$/C.

Supplementary Note 17 | AC-TEM revealed distinct modes of uniform Ru nanocrystal dispersion in UP-$RuNi_{SAs}$/C. Additionally, the high-resolution STEM images displayed the (002) and (101) crystal planes of Ru nanocrystals, further demonstrating its crystal properties of UP-$RuNi_{SAs}$/C.

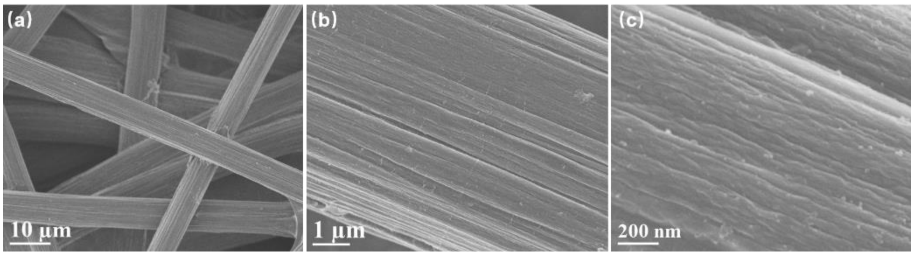

Supplementary Fig. 25 | a, b, c, SEM image of blank carbon paper.

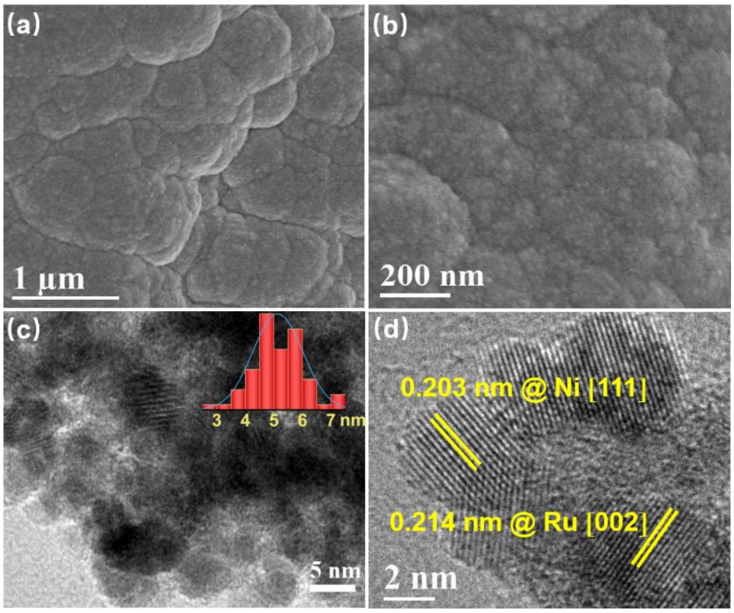

Supplementary Fig. 26 | a,b, SEM, **c**,TEM image (illustration: diameter histogram of CP-RuNi/C), **d**, HRTEM image of CP-RuNi/C.

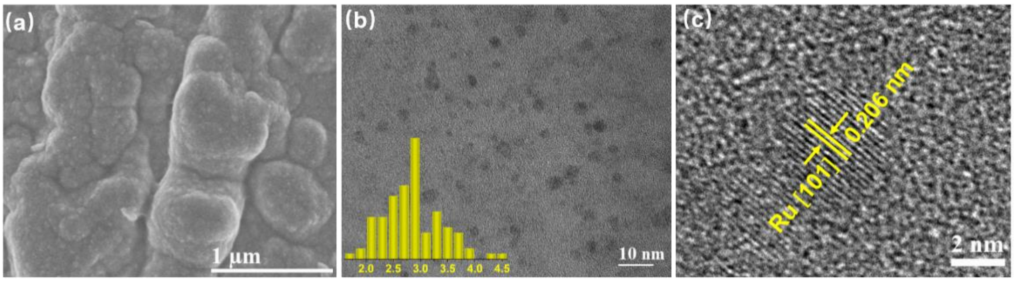

Supplementary Fig. 27 | a, SEM, **b**, TEM image (illustration: diameter histogram of UP-Ru/C), **c**, HRTEM image of UP-Ru/C.

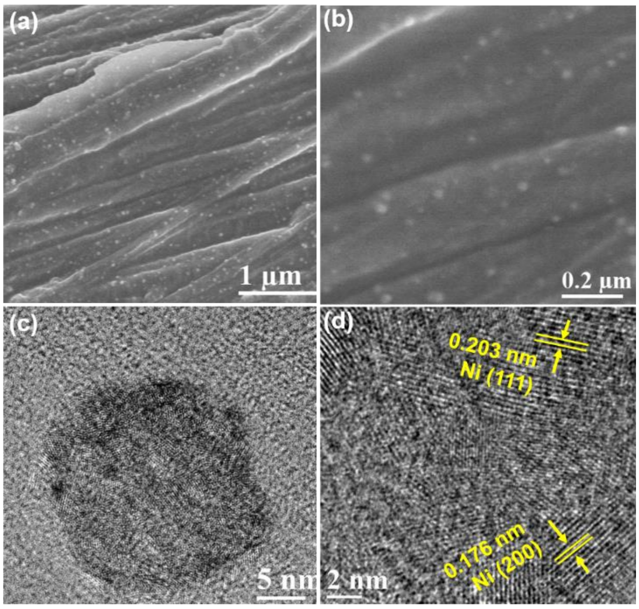

Supplementary Fig. 28 | a,b, SEM, **c**,TEM image, **d**, HRTEM image of UP-Ni/C.

Supplementary Note 18 | The results show that the surface of the UP-Ni/C catalyst exhibits the morphology of nanoparticles, and the corresponding nanocrystals exposed the (200) and (111) crystal planes of Ni, proving that Ni in the catalyst is nanocrystals.

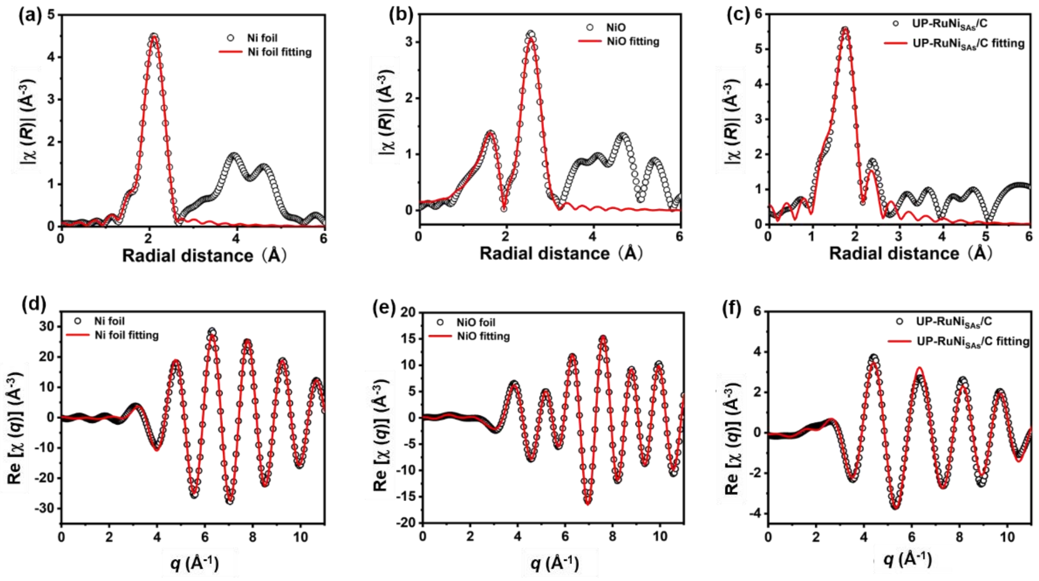

Supplementary Fig. 29 |. **a**, **b**, **c**, R space and **d**, **e**, **f**, inverse FT-EXAFS fitting results of Ni K-edge for **a**,**d**, Ni foil, **b**, **e**, NiO and **c**, **f**, UP-RuNi$_{SAs}$/C.

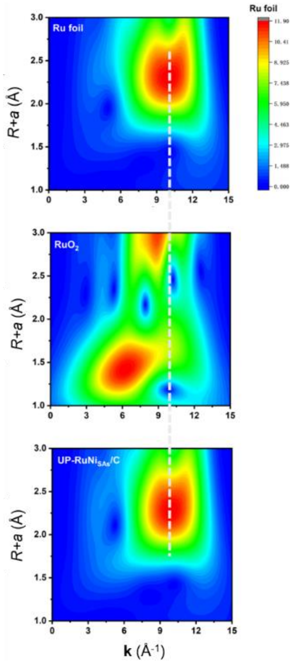

Supplementary Fig. 30 | Wavelet transforms for the k^2-weighted EXAFS signals of UP-$RuNi_{SAs}/C$ RuO_2 and Ru foil.

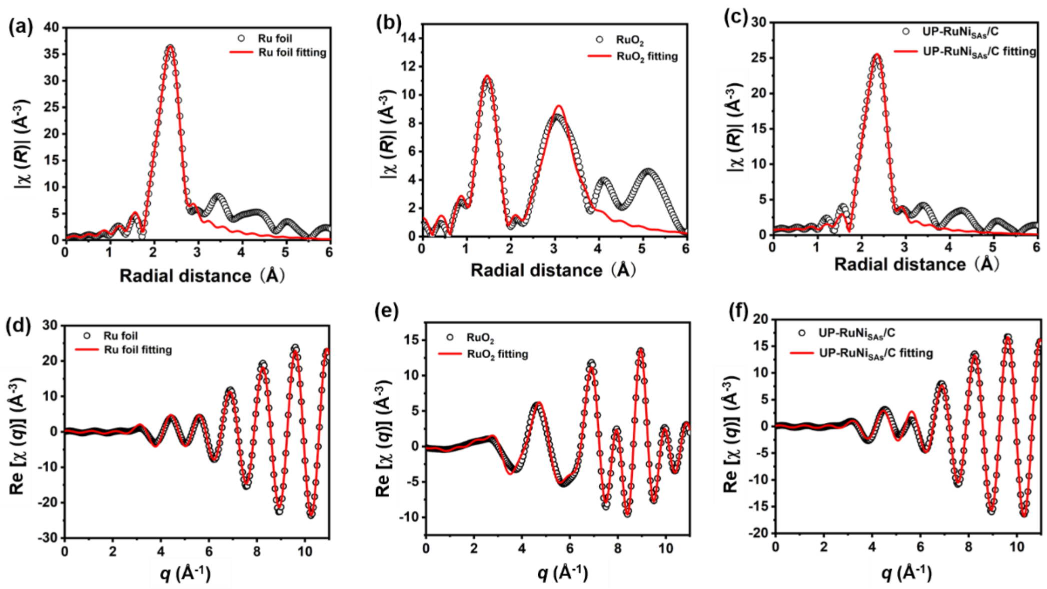

Supplementary Fig. 31 | a, **b**, **c**, R space and **d**, **e**, **f**, inverse FT-EXAFS fitting results of Ru K-edge for **a**, **d**, UP-$RuNi_{SAs}$/C, **b**, **e**, Ru foil and **c**, **f**, RuO_2.

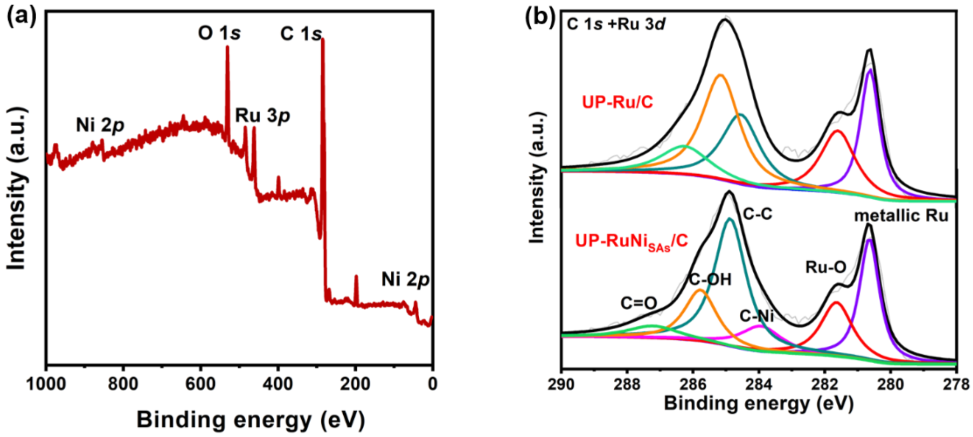

Supplementary Fig. 32 | a, The XPS full spectra of UP-RuNi$_{SAs}$/C catalyst; **b**, the high-resolution XPS spectra of C 1*s* and Ru 3*d* for UP-Ru/C and UP-RuNi$_{SAs}$/C catalysts.

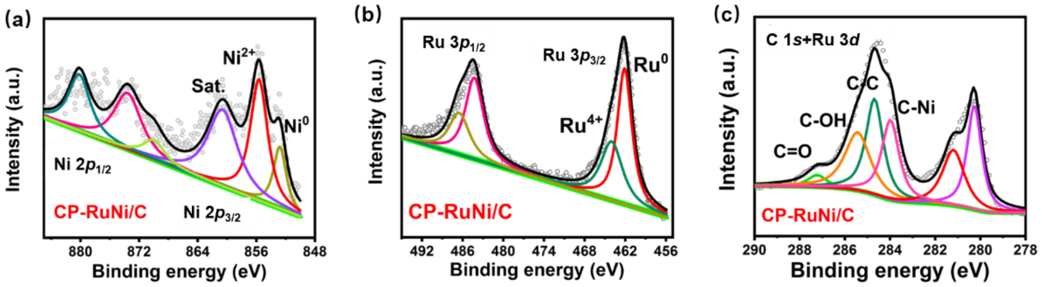

Supplementary Fig. 33 | The high-resolution XPS spectra of **a**, Ni 2*p*, **b**, Ru 3*p* and **c**, C 1*s* and Ru 3*d* for CP-RuNi/C catalyst.

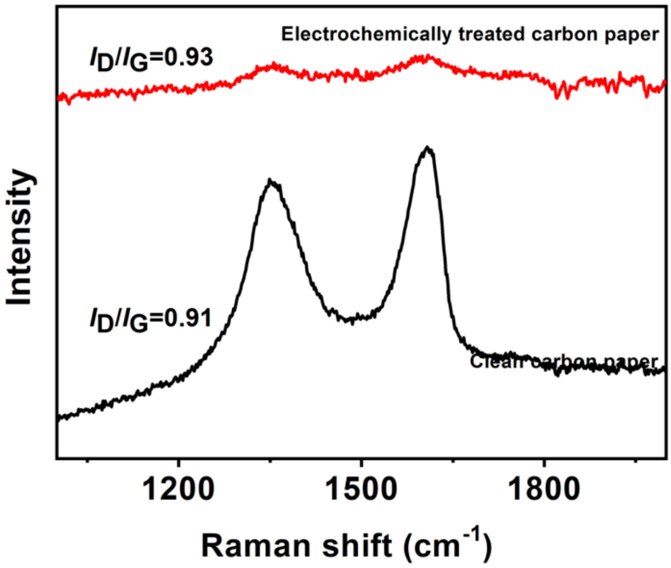

Supplementary Fig. 34 | Raman spectra of clean carbon paper and electrochemically treated carbon paper.

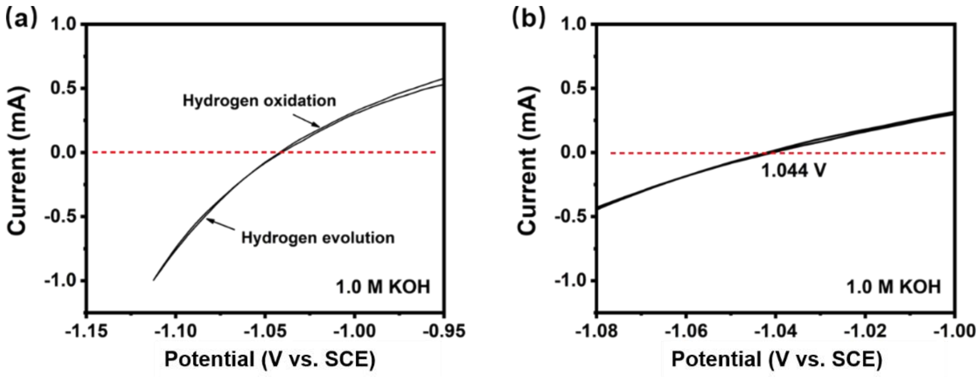

Supplementary Fig. 35 | Saturated calomel electrode (SCE) calibrations in 1.0 M KOH solution, **a**, and **b**, CV curve from -0.95 V to -1.15 V (vs. SCE).

Supplementary Note 19 | The calibration of SCE was achieved in H_2 saturated 1 M KOH solution with a Pt column as both working and counter electrode. Th CV curve was obtained at a scan rate of 1 mV/s. The average potential of H_2 evolution/oxidation under zero current is the potential relative to the standard hydrogen electrode (E_{RHE}). E_{RHE}=E_{SCE}+1.044, in which the pH is 13.56. So the standard electrode potential for SCE in this work is 0.244V.

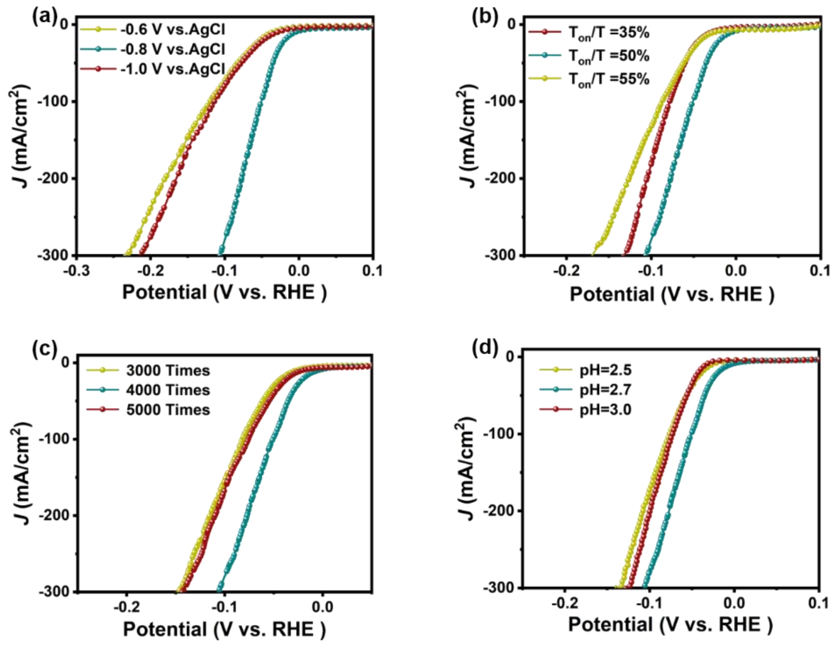

Supplementary Fig. 36 |. Polarization curves of the conditional exploration of **a**, deposition potential, **b**, duty cycle, **c**, number of pulses, **d**, pH for the UPED process of the UP-RuNi$_{SAs}$/C.

Supplementary Note 20 | The polarization curves of the catalysts obtained by UPED for condition exploration were listed in Supplementary Fig. 36. the optimal UPED conditions are: deposition potential is -0.8 V Ag/AgCl, duty cycle is 50%, number of pulses is 4000 times, and pH 2.7 of the deposition solution.

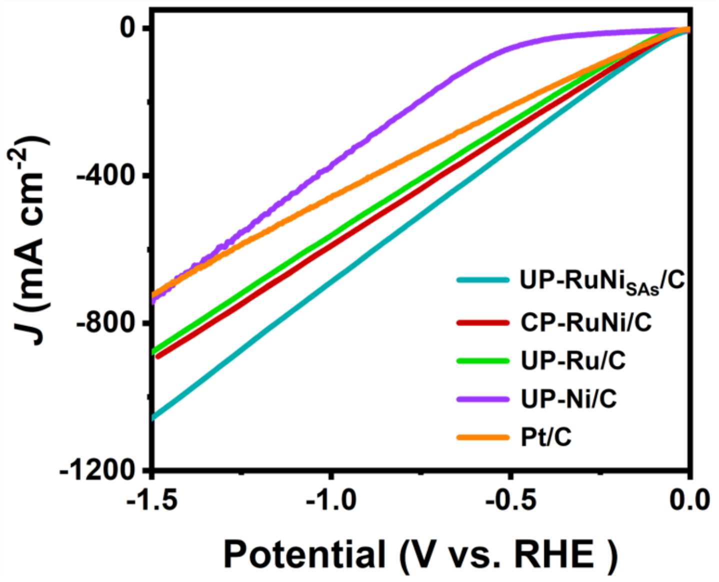

Supplementary Fig. 37 | Polarization curves without iR-compensated of the UP-Ni/C, UP-Ru/C, CP-RuNi/C, UP-RuNi$_{SAs}$/C and commercial Pt/C catalysts in 1.0 M KOH.

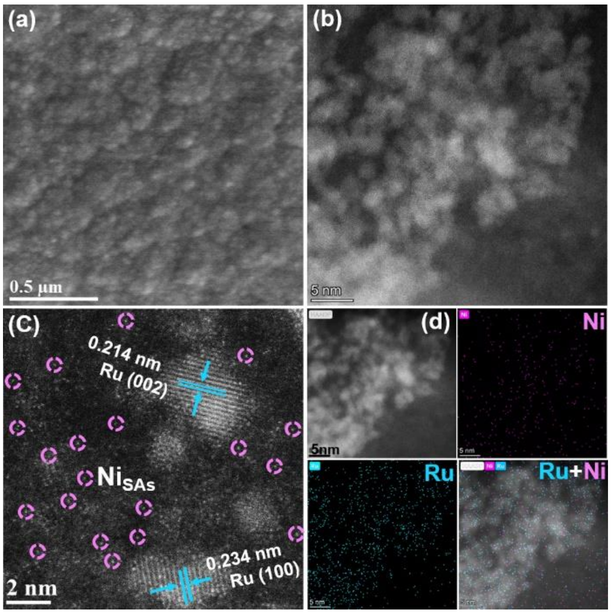

Supplementary Fig. 38 | a, SEM, **b-c**, Spherical aberration correction STEM, in which the bright spots highlighted by pink circles are ascribed to Ni single atoms. **d**, STEM-EDS mapping of UP-RuNi$_{SAs}$/Ti.

Supplementary Note 21 | AC-TEM revealed uniform Ru nanocrystal dispersion in UP-RuNi$_{SAs}$/Ti. Additionally, the high-resolution STEM images displayed the (002) and (100) crystal planes of Ru nanocrystals and dispersed Ni$_{SAs}$.

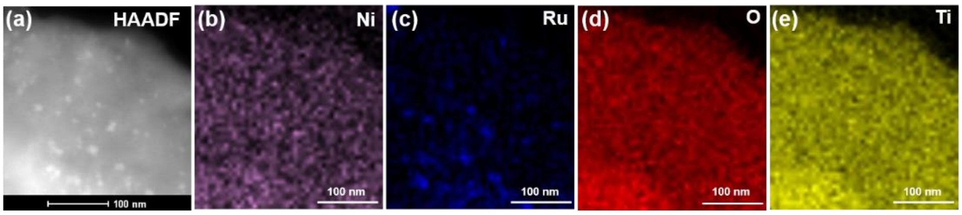

Supplementary Fig. 39 | TEM-EDS mapping of UP-RuNi$_{SAs}$/Ti. (a) STEM, (b-e) EDS mapping images of UP-RuNi$_{SAs}$/Ti.

Supplementary Note 22 | The homogeneous distribution of the four elements and especially the homogeneously dispersed positions of O, Ti and Ni roughly demonstrate the anchoring of Ni$_{SAs}$ on the Ti substrate.

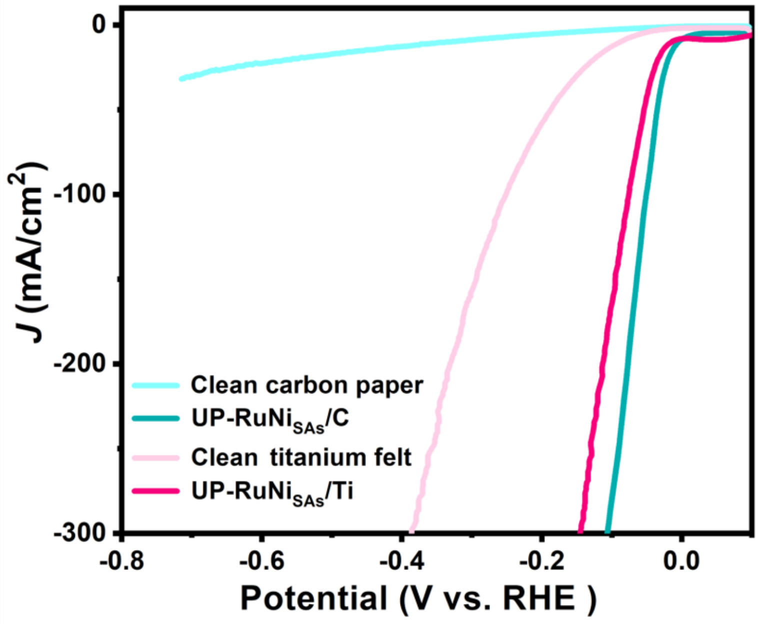

Supplementary Fig. 40 |. Polarization curves of the clean carbon paper, UP-RuNi$_{SAs}$/C, clean titanium felt and UP-RuNi$_{SAs}$/Ti.

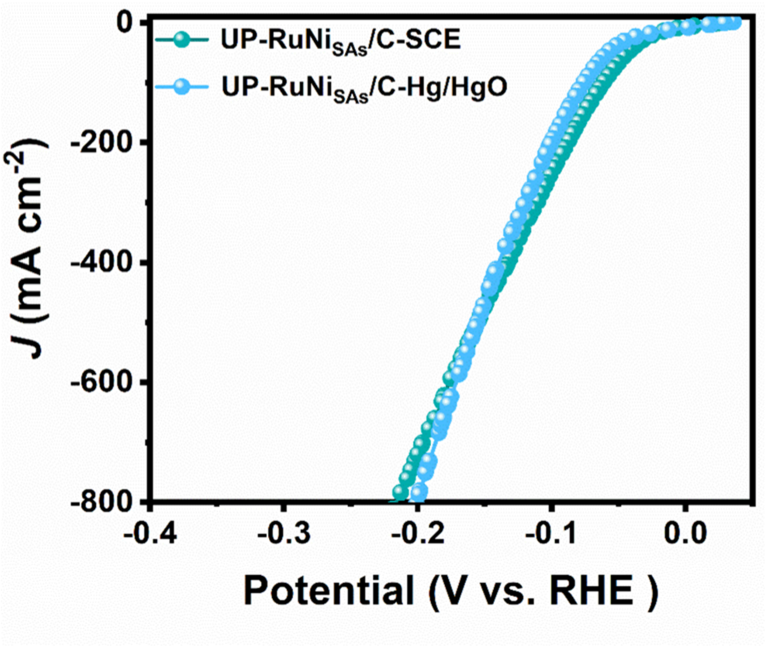

Supplementary Fig. 41 | Polarization curves of UP-RuNi$_{SAs}$/C catalysts with the SCE and Hg/HgO as reference electrodes respectively in 1.0 M KOH.

Supplementary Note 23 | The results show that the two curves almost coincide. Furthermore, the overpotential of UP-RuNi$_{SAs}$/C measured by the Hg/HgO electrode at 10 mA cm^{-2} was 10 mV, which was only 1 mV different from the results measured by the SCE. Therefore, considering the allowable range of error, it can be concluded that the test results with the SCE as reference electrode are accurate.

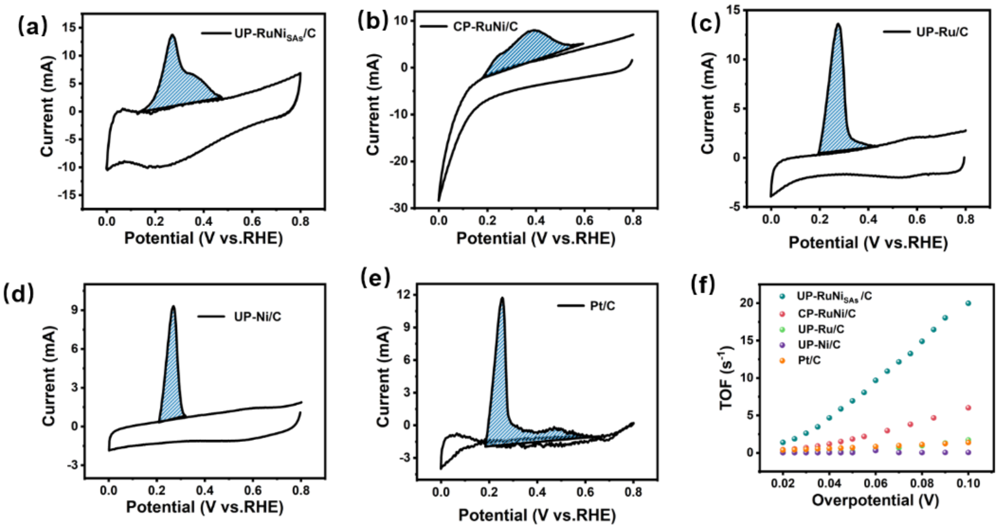

Supplementary Fig. 42 | Copper UPD in 0.5 M H_2SO_4 + 5 mM $CuSO_4$ on **a**, UP-RuNi$_{SAs}$/C, **b**, CP-RuNi/C, c UP-Ru/C, **d**, UP-Ni/C and **e**, Pt/C polarized to form the UPD layers. **f**, TOF values of HER of UP-RuNi$_{SAs}$/C, CP-RuNi/C, UP-Ru/C, UP-Ni/C and Pt/C catalysts.

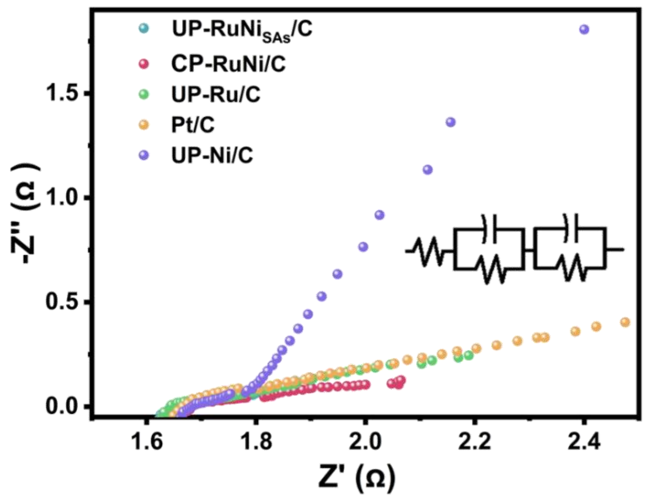

Supplementary Fig. 43 | EIS Nyquist plots of UP-Ni/C, UP-Ru/C, CP-RuNi/C, UP-$RuNi_{SAs}$/C and Pt/C catalysts during HER process under 1.0 M KOH solution.

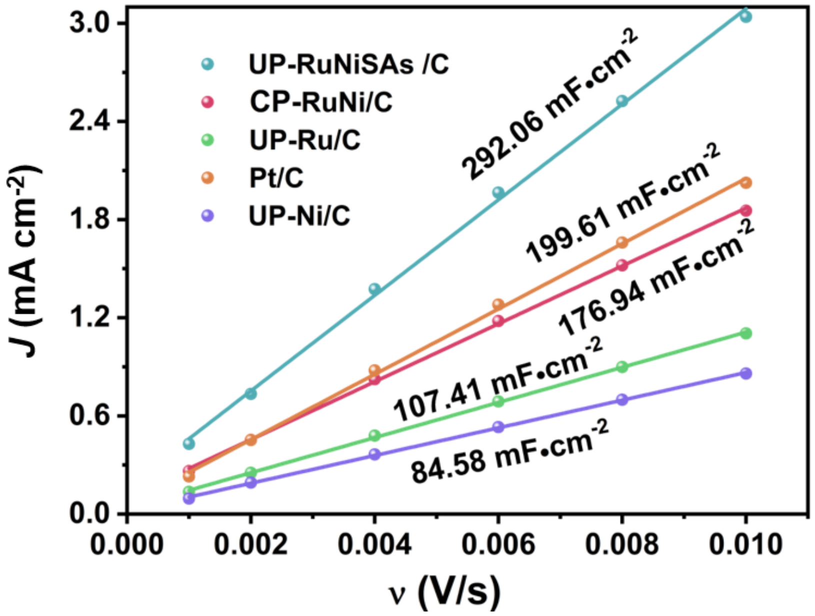

Supplementary Fig. 44 | Capacitive currents against scan rate and corresponding C_{dl} value of UP-Ni/C, UP-Ru/C, CP-RuNi/C, UP-$RuNi_{SAs}$/C and Pt/C catalysts during HER process under 1.0 M KOH solution.

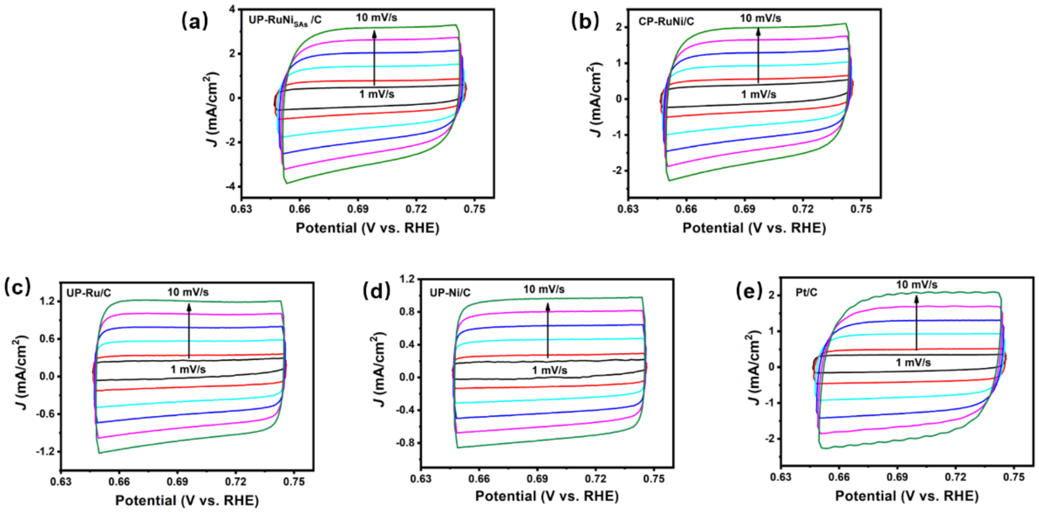

Supplementary Fig. 45 |. The CV curves at different scan rates (1-10 mV·s^{-1}) of **a**, UP-RuNi$_{SAs}$/C, **b**, CP-RuNi/C, **c**, UP-Ru/C, **d**, UP-Ni/C and **e**, Pt/C catalysts during HER process under 1.0 M KOH solution.

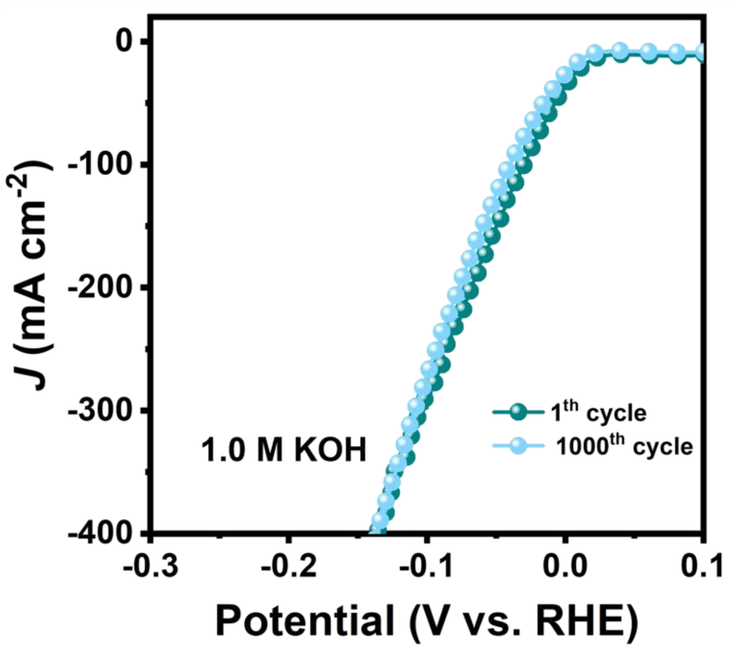

Supplementary Fig. 46 | The polarization curves of UP-RuNi$_{SAs}$/C before and after 1000 cycles CV.

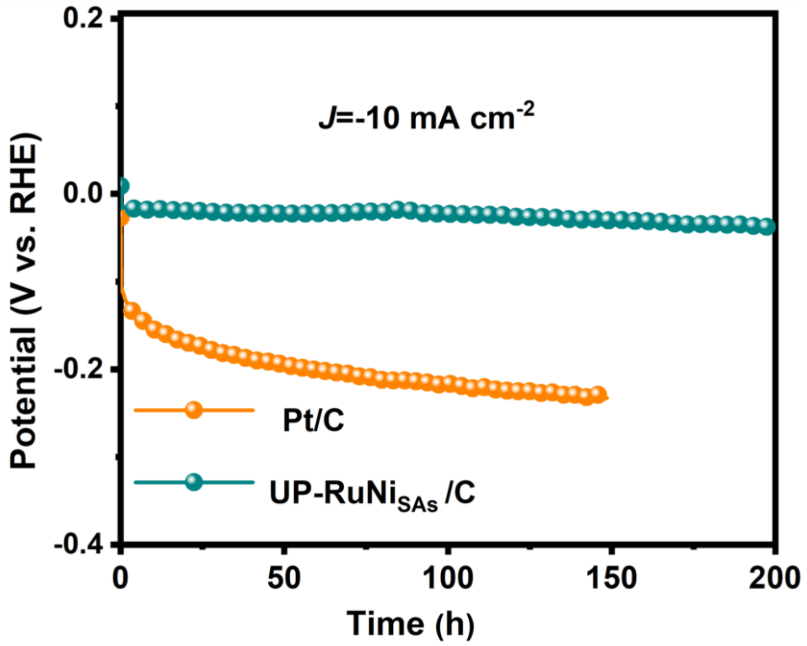

Supplementary Fig. 47 | Long-term stability test of the UP-RuNi$_{SAs}$/C and Pt/C catalysts in 1.0 M KOH.

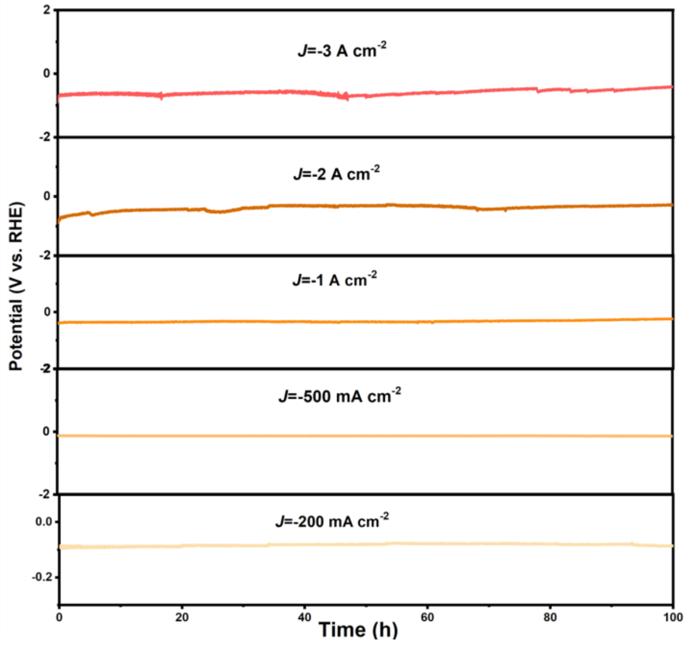

Supplementary Fig. 48 |. Long-term stability test of the UP-$RuNi_{SAs}$/C catalysts in 1.0 M KOH.

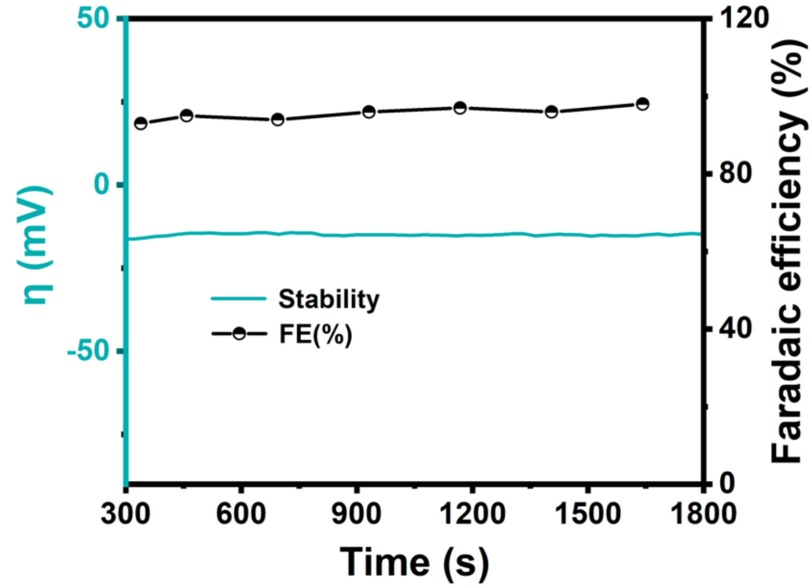

Supplementary Fig. 49 |. Chronopotentiometric curve of UP-RuN_{SAs}/C with constant current density of -10 mA/cm^2 in 1 M KOH, and the corresponding Faradaic efficiency of evolved H_2.

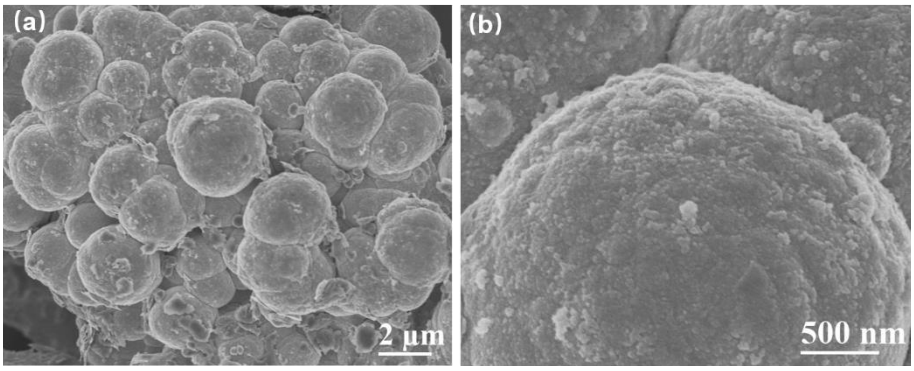

Supplementary Fig. 50 | a-b, SEM images of the UP-$RuNi_{SAs}$/C catalyst after HER test in 1.0 M KOH.

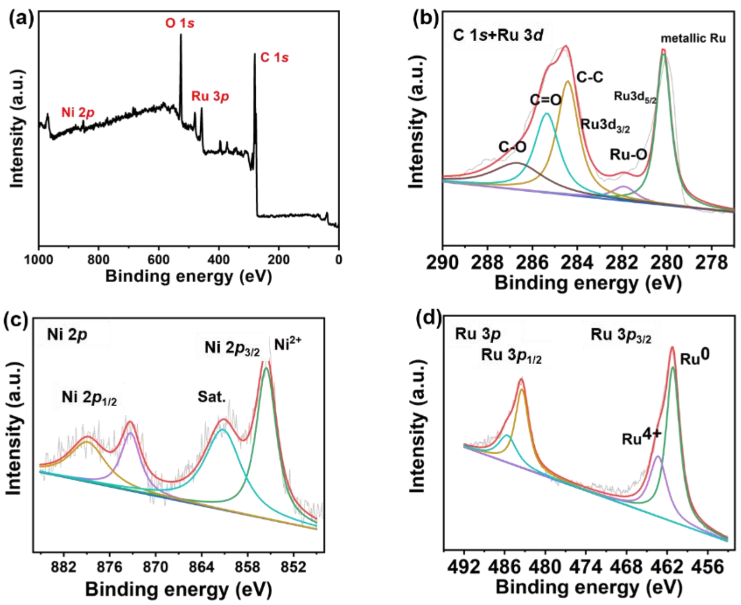

Supplementary Fig. 51 | a, The XPS full spectra, **b**, the high-resolution XPS spectra of C 1*s* and Ru 3*d* **c**, Ni 2*p* and **d**, Ru 3*p* of the UP-$RuNi_{SAs}$/C catalyst after HER test in 1.0 M KOH.

Supplementary Note 24 | After long-term stability testing (100 h), the atomic ratio of each element in the UP-$RuNi_{SAs}$/C is Ru/Ni/C=5.43 at%/1.43 at %/93.14 at %. After 50 h and 100 h of testing, the electrolyte's ICP-OES analysis detected the Ni content to be -0.048mg/L and -0.045mg/L, respectively, indicating that there was virtually no presence of Ni. This finding provides further evidence of the exceptional stability of Ni in UP-$RuNi_{SAs}$/C, as it did not dissolve during long-term stability testing.

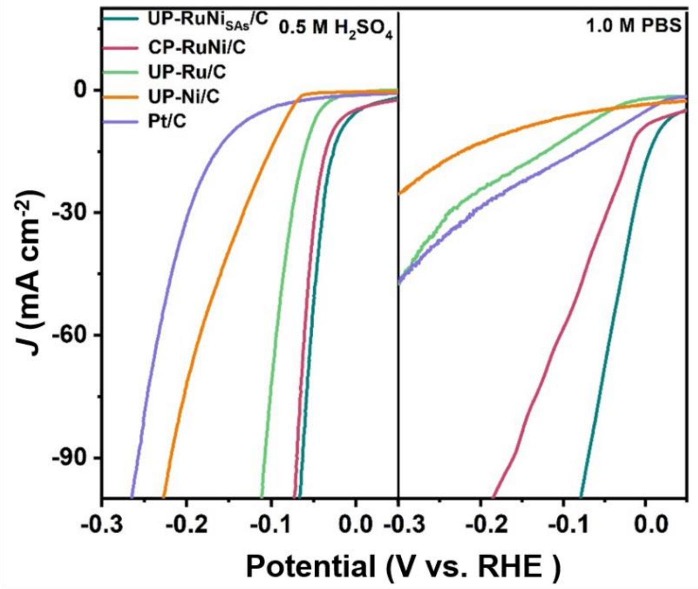

Supplementary Fig. 52 | Polarization curves of the UP-Ni/C, UP-Ru/C, CP-RuNi/C, UP-RuNi$_{SAs}$/C and Pt/C catalysts in 0.5 M H_2SO_4 and 1.0 M PBS solution.

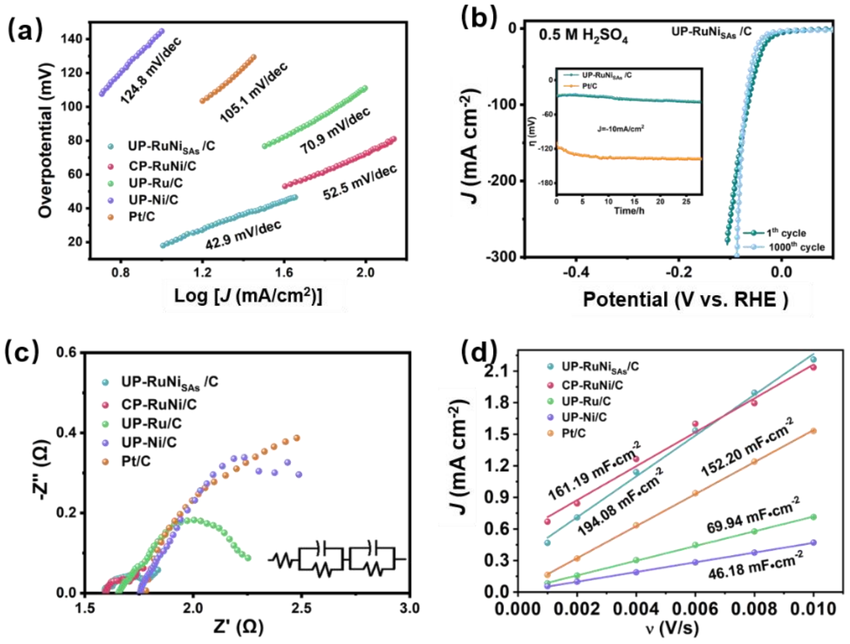

Supplementary Fig. 53 | a, Tafel plots of the UP-Ni/C, UP-Ru/C, CP-RuNi/C, UP-$RuNi_{SAs}$/C and Pt/C catalysts, **b**, cyclic stability of UP-$RuNi_{SAs}$/C and long-term stability test of the UP-$RuNi_{SAs}$/C and Pt/C catalysts, **c**, EIS Nyquist plots, **d**, capacitive currents against scan rate and corresponding C_{dl} value of UP-Ni/C, UP-Ru/C, CP-RuNi/C, UP-$RuNi_{SAs}$/C and Pt/C catalysts during HER process under 0.5 M H_2SO_4 solution.

Supplementary Note 25 | The UP-$RuNi_{SAs}$/C presented favorable HER performance in acidic conditions among the other catalysts for contrast, with the lowest Tafel slope, minimum charge transfer internal resistance (R_{ct}) and largest smallest calculated double layer capacitance (C_{dl}) value. In addition, the chronopotentiometry test at the current density of -10 mA cm^{-2} for UP-$RuNi_{SAs}$/C shown that it maintained absolute stability within 27 hours, superior to the commercial Pt/C electrocatalyst with the stability continuous attenuation in 0.5 M H_2SO_4 solution.

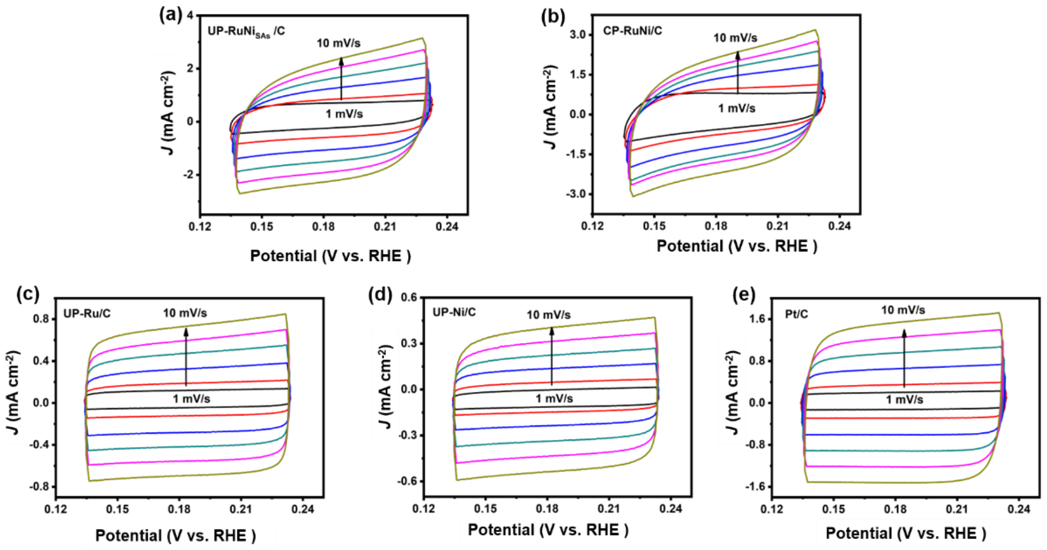

Supplementary Fig. 54 | The CV curves at different scan rates (1-10 mV·s^{-1}) of **a**, UP-RuNi$_{SAs}$/C, **b**, CP-RuNi/C, **c**, UP-Ru/C, **d**, UP-Ni/C and **e**, Pt/C catalysts during HER process under 0.5 M H_2SO_4 solution.

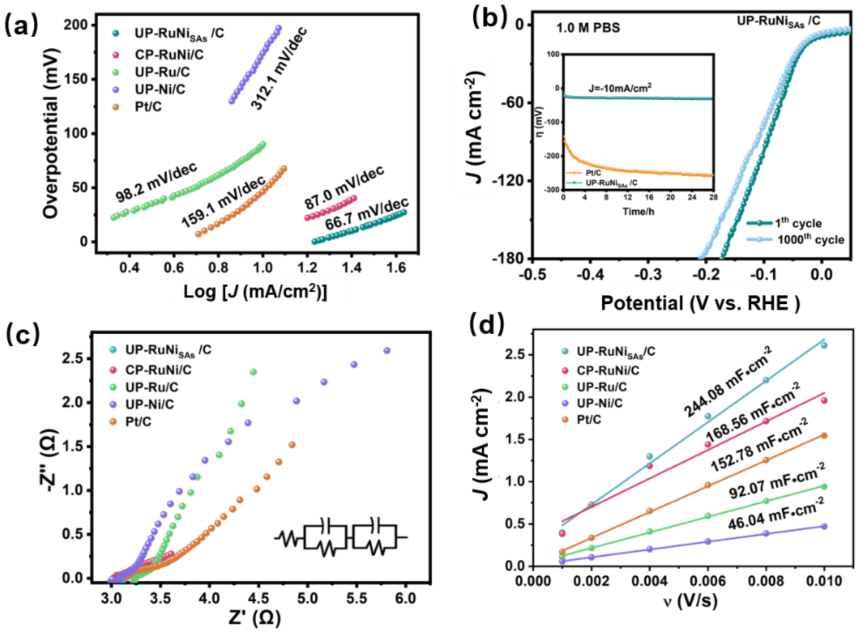

Supplementary Fig. 55 | a, Tafel plots of the UP-Ni/C, UP-Ru/C, CP-RuNi/C, UP-$RuNi_{SAs}$/C and Pt/C catalysts, **b**, cyclic stability of UP-$RuNi_{SAs}$/C and long-term stability test of the UP-$RuNi_{SAs}$/C and Pt/C catalysts, **c**, EIS Nyquist plots, **d**, capacitive currents against scan rate and corresponding C_{dl} value of UP-Ni/C, UP-Ru/C, CP-RuNi/C, UP-$RuNi_{SAs}$/C and Pt/C catalysts during HER process under 1.0 M PBS solution.

Supplementary Note 26 | The UP-$RuNi_{SAs}$/C presented favorable HER performance in neutral conditions among the other catalysts for contrast, with the lowest Tafel slope, minimum charge transfer internal resistance (R_{ct}) and largest smallest calculated double layer capacitance (C_{dl}) value. In addition, the chronopotentiometry test at the current density of -10 mA cm^{-2} for UP-$RuNi_{SAs}$/C shown that it maintained absolute stability within 28 hours, superior to the commercial Pt/C electrocatalyst with the stability continuous attenuation in1.0 M PBS solution.

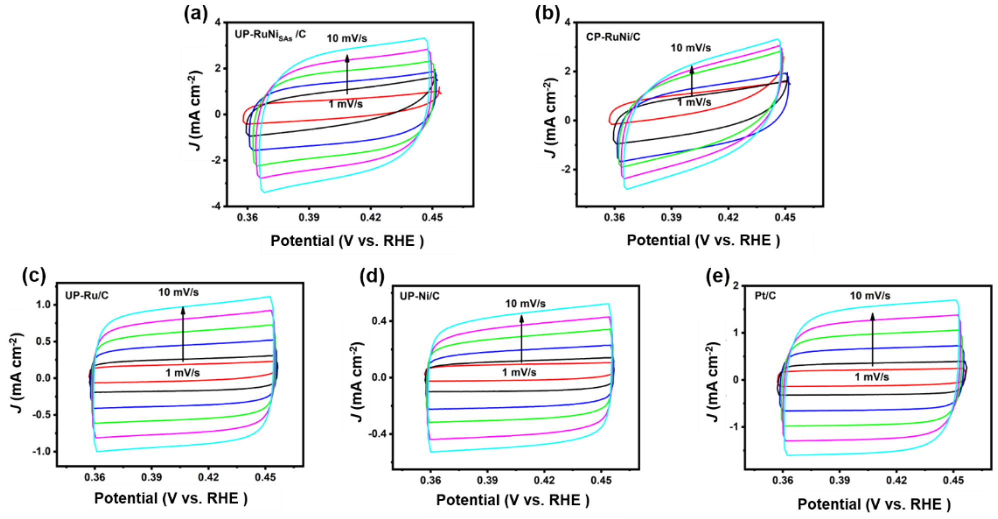

Supplementary Fig. 56 |. The CV curves at different scan rates (1-10 mV·s^{-1}) of **a**, UP-RuNi$_{SAs}$/C, **b**, CP-RuNi/C, **c**, UP-Ru/C, **d**, UP-Ni/C and **e**, Pt/C catalysts during HER process under 1.0 M PBS solution.

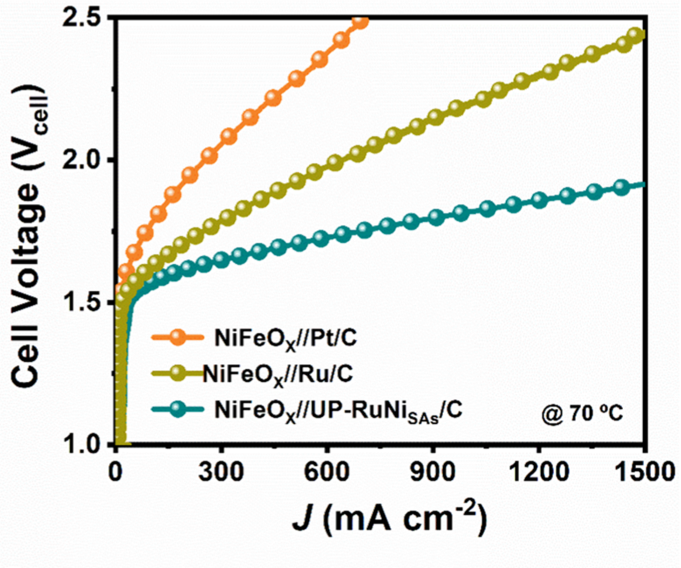

Supplementary Fig. 57 | LSV curves of the AEM reactors using $NiFeO_x$ as the anodic and UP-$RuNi_{SAs}$/C, commercial Pt/C and Ru/C as the cathodic electrodes, respectively at 70 °C

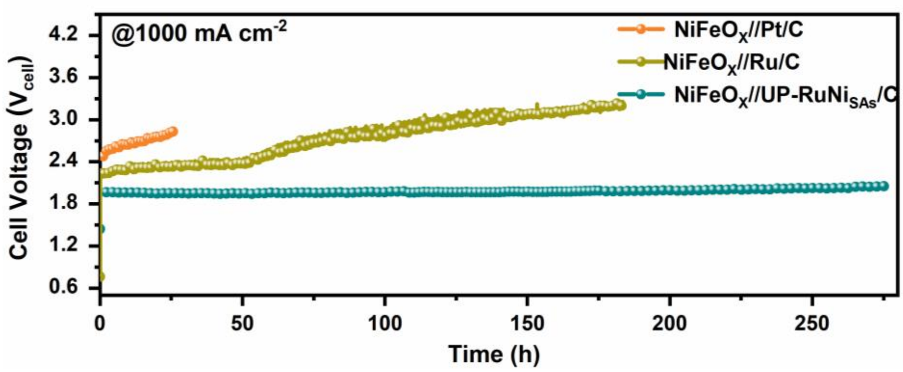

Supplementary Fig. 58 | Stability tests of the AEM water electrolysers at 1 A cm^{-2} using $NiFeO_x$ as the cathodic and UP-$RuNi_{SAs}$/C, commercial Pt/C and Ru/C as the anodic electrodes, respectively at 70 °C

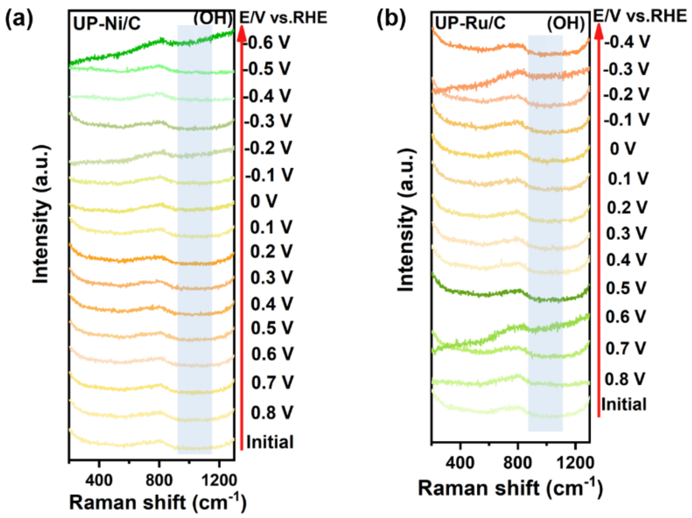

Supplementary Fig. 59 | Operando Raman spectroscopy experiments for **a**, UP-Ni/C and **b**, UP-Ru/C.

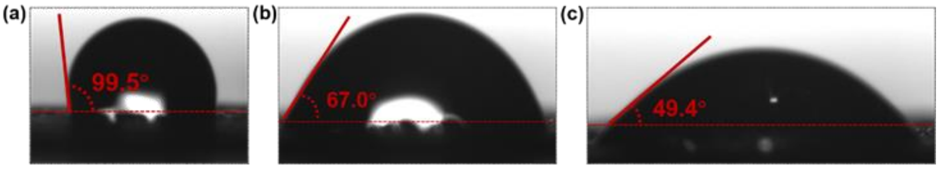

Supplementary Fig. 60 | Contact angle measurement for **a**, UP-Ru/C, **b**, CP-RuNi/C, **c**, UP-$RuNi_{SAs}$/C electrocatalysts.

Supplementary Tables

Supplementary Table 1 | Adsorption energy of various intermediate adsorption (OH^* and H^*) on active sites for HER corresponding to different models.

Model	E_{OH^*} (eV)	$\lvert E_{H^*} \rvert$ (eV)
Ru (002)	-0.43	0.53
Ni (111)	-0.25	0.71
Ni_{sub}	0.29	0.72
Ni_{def}	0.32	0.55
$RuNi_{sub}$/C	0.35	0.24
$RuNi_{def}$/C	-0.15	0.15

Supplementary Table 2 | Exploration of the effect of the deposition potential on the UPED process of the UP-$RuNi_{SAs}$/C.

Pulsed deposition potential	Ru(wt.%)	Ni(wt.%)
-0.5 V vs. Ag/AgCl	98.267	1.733
-0.8 V vs. Ag/AgCl	99.012	0.988
-1.0 V vs. Ag/AgCl	92.607	7.393
-1.2 V vs. Ag/AgCl	60.652	39.348

Supplementary Table 3 | Exploration of the effect of the duty cycle (T_{on}/T) on the UPED process of the UP-$RuNi_{SAs}$/C.

T_{on}/T (%)	Ru(wt.%)	Ni(wt.%)
75	96.885	3.115
55	97.966	2.034
50	99.012	0.988
35	91.261	8.739
25	92.775	7.225
15	98.137	1.863

Supplementary Table 4 | Exploration of the effect of the number of pulses on the UPED process of the UP-RuNi$_{SAs}$/C.

**Number of pulses（Times）**	**Ru(wt.%)**	**Ni(wt.%)**
2000	91.347	3.115
3000	93.952	2.034
4000	99.012	0.988
5000	98.974	1.026

Supplementary Table 5 | EXAFS fitting parameters at the Ni K-edge for various samples (S_0^2=1.0).

Sample	Shell	N[a]	R(Å)[b]	σ^2(Å^2)[c]	S_0^2	ΔE_0(eV)[d]	R factor
**Ni foil**	Ni-Ni	12	2.48	0.0078	1.0	6.30	0.0103
**NiO**	Ni-O	6	2.09	0.0076	1.0	2.84	0.0059
	Ni-Ni	12	2.96	0.0064	1.0	9.97	
**UP-**	Ni-C	3	2.00	0.0061	1.0	6.42	0.0125
**RuNi$_{SAs}$/C**	Ni-O	0.4	2.14	0.0075	1.0	5.45	

Supplementary Note 27 | [a]CN, coordination number; [b]R, distance between absorber and backscatter atoms; [c]σ^2, Debye-Waller factor to account for both thermal and structural disorders; [d]ΔE_0, inner potential correction; R factor indicates the goodness of the fit. According to the experimental EXAFS fit of Ni foil by fixing CN as the known crystallographic value. Fitting range: 3.0 ≤ k (/Å) ≤ 11.8 and 1.0 ≤ R (Å) ≤ 3.0 (Ni foil); 3.0≤ k (/Å) ≤ 11.8 and 1.0 ≤ R (Å) ≤ 3.0 (NiO).; 3.0 ≤ k (/Å) ≤ 11.2 and 1.0 ≤ R (Å) ≤ 3.1 (UP-RuNi$_{SAs}$/C).

Supplementary Table 6 | EXAFS fitting parameters at the Ru K-edge for various samples (S_0^2=1).

Sample	Path	N^a	R(Å)^b	σ^2(Å^2)^c	ΔE_0(eV)^d	*R* factor
**Ru foil**	Ru-Ru	6.00	2.67	0.0034	-4.24	0.0070
**RuO_2**	Ru-O	3.57	1.97	0.0027	-0.159	0.0115
	Ru-Ru1	0.94	3.56	0.0055		
	Ru-Ru2	2.72	3.14	0.0040		
**UP-$RuNi_{SAs}$/C**	Ru-Ru1	1.81	2.52	0.0029	4.99	0.0100
	Ru-Ru2	1.79	2.71	0.0020		

Supplementary Note 28 | [a]CN, coordination number; [b]*R*, distance between absorber and backscatter atoms; [c]σ^2, Debye-Waller factor to account for both thermal and structural disorders; [d]ΔE_0, inner potential correction; *R* factor indicates the goodness of the fit. According to the experimental EXAFS fit of Ru foil by fixing CN as the known crystallographic value. Fitting range: 2.5 ≤ *k* (/Å) ≤ 11.6 and 1.0 ≤ *R* (Å) ≤ 2.3 (Ru foil).; 3.0≤*k* (/Å) ≤ 11.3 and 1.0 ≤ *R* (Å) ≤ 3.7(RuO_2).3.0 ≤ *k* (/Å) ≤ 12.7 and 1.3 ≤ *R* (Å) ≤ 3.3 (UP-$RuNi_{SAs}$/C).

Supplementary Table 7 | Comparison of TOF values for other recently reported electrocatalysts in alkaline media.

Catalysts	Electrolyte	Overpotential (mV)	TOF (H_2 s^{-1})	Reference
UP-$RuNi_{SAs}$/C	1.0 M KOH	9 mV	6.86 at 50 mV	This work
Ru-Mo_2S/CC	1.0 M KOH	41 mV	0.2 at 169 mV	[1]
$Ni_{cluster}$-Ru	1.0 M KOH	17 mV	8.95 at 50 mV	[2]
W-ACs	1.0 M KOH	53 mV	0.12 at 50 mV	[3]
Ru-NBC-1	1.0 M KOH	14 mV	0.39 at 25 mV	[4]
Ru-NBC-1	1.0 M KOH	14 mV	1.12 at 100 mV	[4]
np-$Cu_{53}Ru_{47}$	1.0 M KOH	15 mV	1.14 at 100 mV	[5]
2DPC-RuMo	1.0 M KOH	18 mV	3.57 at 100 mV	[6]
C-Co_2P	1.0 M KOH	30 mV	0.14 at 100 mV	[7]

Supplementary Table 8 | Rs, Rct values corresponding to EIS Nyquist fitting circuit diagram of UP-Ni/C, UP-Ru/C, CP-RuNi/C, UP-RuNi$_{SAs}$/C catalysts during HER process under 1.0 M KOH solution.

**Samples**	**R_S (Ω)**	**$R_{ct,1}$ (Ω)**	**$R_{ct,2}$ (Ω)**
UP-RuNi$_{SAs}$/C	1.613	0.1021	0.1236
CP-RuNi/C	1.712	0.1252	0.2436
UP-Ru/C	1.976	0.1654	0.4389
UP-Ni/C	1.714	0.1439	9.344

Supplementary Table 9 | Rs, Rct values corresponding to EIS Nyquist fitting circuit diagram of UP-Ni/C, UP-Ru/C, CP-RuNi/C, UP-RuNi$_{SAs}$/C catalysts during HER process under 0.5 M H_2SO_4 solution.

**Samples**	**R_S (Ω)**	**$R_{ct,1}$ (Ω)**	**$R_{ct,2}$ (Ω)**
UP-RuNi$_{SAs}$/C	1.614	0.1119	0.0870
CP-RuNi/C	1.627	0.0780	0.1124
UP-Ru/C	1.685	0.3940	0.1386
UP-Ni/C	1.787	0.7235	0.1771

Supplementary Table 10 | R_s, R_{ct} values corresponding to EIS Nyquist fitting circuit diagram of UP-Ni/C, UP-Ru/C, CP-RuNi/C, UP-$RuNi_{SAs}$/C catalysts during HER process under 1.0 M PBS solution.

**Samples**	**R_S (Ω)**	**$R_{ct,1}$ (Ω)**	**$R_{ct,2}$ (Ω)**
UP-$RuNi_{SAs}$ /C	3.170	0.3611	0.1649
CP-RuNi/C	3.065	0.4729	0.1920
UP-Ru/C	3.295	0.2360	5.497
UP-Ni/C	4.076	4.579	0.2781

Supplementary Table 11 | Comparison of various electrocatalysts for HER.

Catalysts	Overpotential at 10 mA cm^{-2} (mV)			Reference
	alkali	acid	neutral	
UP-$RuNi_{SAs}$/C	9	18	27	This work
NiCoP/Mo_xC	79	116	-	[8]
$RhSe_2$	81.6	49.9	-	[9]
RuCo@CDs	11	51	67	[10]
N-Co_2P	34	27	42	[11]
Ni_5P_4-Ru	54	-	-	[12]
$NiRu_{0.13}$-BDC	34	13	36	[13]
Ru/OMSNNC	13	27	70	[14]
h-$RuSe_2$	34	-	-	[15]
Ru_1CoP/CD_s	51	49	-	[16]
Au@$AuIr_2$	-	29	-	[17]
Ni-Pt	-	90	-	[18]
Ru/RuS_2-2		45		[19]
Ru-NiCoP/NF	44	-	-	[20]
IrCo-NPs		24		[21]
Ru/Ni_2P@NPC	132	89	124	[22]

Supplementary References：

[1] Wang D, Li Q, Han C, Xing Z, Yang X. Single-atom ruthenium based catalyst for enhanced hydrogen evolution. *Appl. Catal. B: Environ.* **249**, 91-97 (2019).

[2] Zhu T, Liu S, Huang B, Shao Q, Wang M, Li F, et al. High-performance diluted nickel nanoclusters decorating ruthenium nanowires for pH-universal overall water splitting. *Energy. Environ. Sci*. **14**, 3194-3202 (2021).

[3] Chen Z, Xu Y, Ding D, Song G, Gan X, Li H, et al. Thermal migration towards constructing WW dual-sites for boosted alkaline hydrogen evolution reaction. Nat. Commun. **13**, 1-12 (2022).

[4] Wu T, Hong J, Lu Z, Wu H, Wu C, Tang Z, et al. In-situ generation of Ru-catechol coordinative polymer precursor for high-performance hydrogen evolution reaction doped carbon catalyst. *Appl. Catal. B: Environ*. **285**, 119795 (2021).

[5] Wu Q, Luo M, Han J, Peng W, Zhao Y, Chen D, et al. Identifying electrocatalytic sites of the nanoporous copper–ruthenium alloy for hydrogen evolution reaction in alkaline electrolyte. ACS Energy Lett. **5**, 192-199 (2019).

[6] Tu K, Tranca D, Rodríguez‐Hernández F, Jiang K, Huang S, Zheng Q, et al. A Novel Heterostructure Based on RuMo Nanoalloys and N‐doped Carbon as an Efficient Electrocatalyst for the Hydrogen Evolution Reaction. *Adv. Mater.* **32**, 2005433 (2020).

[7] Xu W, Fan G, Zhu S, Liang Y, Cui Z, Li Z, et al. Electronic structure modulation of nanoporous cobalt phosphide by carbon doping for alkaline hydrogen evolution reaction. *Adv. Funct. Mater.* **31**, 2107333 (2021).

[8] Wang Y, Wang B, Chu W, Kong Y, Wu Q, Liu Z. Engineering NiCoP/MoxC heterojunction for highly efficient hydrogen evolution reaction in alkaline and acid solution. *Int. J. Hydrogen Energ.* **45**, 28774-28782 (2020).

[9] Zhong W, Xiao B, Lin Z, Wang Z, Huang L, Shen S, et al. RhSe2: A superior 3D electrocatalyst with multiple active facets for hydrogen evolution reaction in both acid and alkaline solutions. Adv. Mater. **33**, 2007894 (2021).

[10] Feng T, Yu G, Tao S, Zhu S, Ku R, Zhang R, et al. A highly efficient overall water splitting ruthenium-cobalt alloy electrocatalyst across a wide pH range via electronic coupling with carbon dots. *J. Mater. Chem. A.* **8**, 9638-9645 (2020).

[11] Men Y, Li P, Zhou J, Cheng G, Chen S, Luo W. Tailoring the electronic structure of Co2P by N doping for boosting hydrogen evolution reaction at all pH values. *ACS Catal.* **9**, 3744-3752 (2019).

[12] He Q, Tian D, Jiang H, Cao D, Wei S, Liu D, et al. Achieving efficient alkaline hydrogen evolution reaction over a Ni5P4 catalyst incorporating single‐atomic Ru sites. *Adv. Mater.* **32**, 1906972 (2020).

[13] Sun Y, Xue Z, Liu Q, Jia Y, Li Y, Liu K, et al. Modulating electronic structure of metal-organic frameworks by introducing atomically dispersed Ru for efficient hydrogen evolution. *Nat. Commun*. **12**, 1-8 (2021).

[14] Wu YL, Li X, Wei YS, Fu Z, Wei W, Wu XT, et al. Ordered macroporous superstructure of nitrogen‐doped nanoporous carbon implanted with ultrafine Ru nanoclusters for efficient pH‐universal hydrogen evolution reaction. *Adv. Mater*. **33**, 2006965 (2021).

[15] Zhao Y, Cong H, Li P, Wu D, Chen S, Luo W. Hexagonal RuSe2 nanosheets for highly efficient hydrogen evolution electrocatalysis. *Angew. Chem. Int. Ed*. **133**, 7089-7093 (2021).

[16] Song H, Wu M, Tang Z, Tse JS, Yang B, Lu S. Single Atom Ruthenium‐Doped CoP/CDs Nanosheets via Splicing of Carbon‐Dots for Robust Hydrogen Production. *Angew. Chem. Int. Ed.* **60**, 7234-7244 (2021).

[17] Wang H, Chen Z-n, Wu D, Cao M, Sun F, Zhang H, et al. Significantly enhanced overall water splitting performance by partial oxidation of Ir through Au modification in core–shell alloy structure. *J. Am. Chem. Soc.* **143**, 4639-4645 (2021).

[18] Eiler K, Suriñach S, Sort J, Pellicer E. Mesoporous Ni-rich Ni–Pt thin films: Electrodeposition, characterization and performance toward hydrogen evolution reaction in acidic media. *Appl. Catal. B: Environ.* **265**, 118597 (2020).

[19] Zhu J, Guo Y, Liu F, Xu H, Gong L, Shi W, et al. Regulative electronic states around ruthenium/ruthenium disulphide heterointerfaces for efficient water splitting in acidic media. *Angew. Chem. Int. Ed.* **133**, 12436-12442 (2021).

[20] Chen D, Lu R, Pu Z, Zhu J, Li H-W, Liu F, et al. Ru-doped 3D flower-like bimetallic phosphide with a climbing effect on overall water splitting. *Appl. Catal. B: Environ.* **279**, 119396 (2020).

[21] Sun X, Liu F, Chen X, Li C, Yu J, Pan M. Iridium-doped ZIFs-derived porous carbon-coated IrCo alloy as competent bifunctional catalyst for overall water splitting in acid medium. *Electrochim. Acta.* **307**, 206-213 (2019).

[22] Chi J-Q, Zhang X-Y, Ma X, Dong B, Zhang J-Q, Guo B-Y, et al. Interface charge engineering of ultrafine Ru/Ni_2P nanoparticles encapsulated in N, P-codoped hollow carbon nanospheres for efficient hydrogen evolution. *ACS Sustainable Chem. Eng.* **71**, 7714-7722 (2019).
